# Temporal Proteomic and Phosphoproteomic Profiling Deciphers Molecular Dynamics of Acute-to-Chronic Kidney Disease After Ischemia-Reperfusion Injury, With Dock2 Emerging as a Key Regulator

**DOI:** 10.1016/j.mcpro.2026.101509

**Published:** 2026-01-12

**Authors:** Shaowu Zhang, Huasheng Luo, Miaotao Wei, Yanmei Yu, Hongluan Wu, Tongtong Ma, Minjie Zhang, Huafeng Liu, Peng Wang

**Affiliations:** 1 Guangdong Provincial Key Laboratory of Autophagy and Major Chronic Non-Communicable Diseases, Key Laboratory of Prevention and Management of Chronic Kidney Diseases of Zhanjiang City, Institute of Nephrology, Affiliated Hospital of Guangdong Medical University, Zhanjiang, Guangdong, China; 2 Dr. Neher’s Biophysics Laboratory for Innovative Drug Discovery, State Key Laboratory of Quality Research in Chinese Medicine, Faculty of Chinese Medicine, Macau University of Science and Technology, Taipa, Macau, China; 3 Department of Anesthesiology, Affiliated Hospital of Guangdong Medical University, Zhanjiang, Guangdong, China

**Keywords:** AKI-to-CKD progression, Dock2, ischemia-reperfusion injury, phosphoproteomic, proteomic

## Abstract

Acute kidney injury (AKI), characterized by a rapid decline in renal function, has high mortality rates and frequently progresses to chronic kidney disease (CKD). A major contributor to AKI is ischemia-reperfusion injury (IRI). However, the global molecular changes underlying the AKI-to-CKD transition post-IRI remain to be fully elucidated. Using 4D label-free proteomic and phosphoproteomic analyses in a murine unilateral IRI model at 1 h, 1 day, 3 days, 7 days, and 28 days post injury, we systematically identified dysregulated proteins, phosphoproteins, and signaling pathways involved in the progression from AKI to CKD. Critically, these analyses consistently revealed the enrichment and sustained activation of NF-κB signaling, a key pathway driving inflammatory and fibrotic responses, across multiple time points. In addition, we identified significant impairment of fatty acid β-oxidation. Notably, our omics analysis specifically identified the dedicator of cytokinesis (Dock) protein family, with Dock2 emerging as a prime candidate due to its known immune regulatory functions. Dock2 expression showed significant upregulation post-IRI and was found predominantly localized to injured tubular epithelial cells. Functional validation demonstrated that Dock2 knockdown attenuated proinflammatory responses in tubular epithelial cells by inhibiting IKKβ-mediated NF-κB activation *in vitro*. Consistently, pharmacological inhibition of Dock2 by CPYPP ameliorated renal tubular injury, inflammation, and fibrosis *in vivo*. To our knowledge, this is the first study to reveal the role and mechanism of Dock2 in the AKI-to-CKD progression post-IRI. In conclusion, our findings delineate molecular mechanisms underpinning the transition from AKI to CKD and nominate Dock2 as a promising therapeutic target for mitigating this process.

## Highlights

• Temporal proteomic and phosphoproteomic analysis reveals molecular dynamics in renal IRI.
• Sustained NF-κB activation and impaired FAO across AKI-to-CKD transition.
• Dock2 mediates proinflammatory responses in TECs through the IKKβ/NF-κB pathway.
• Targeting Dock2 mitigates AKI-to-CKD progression after IRI.

## Graphical Abstract

**Figure:**
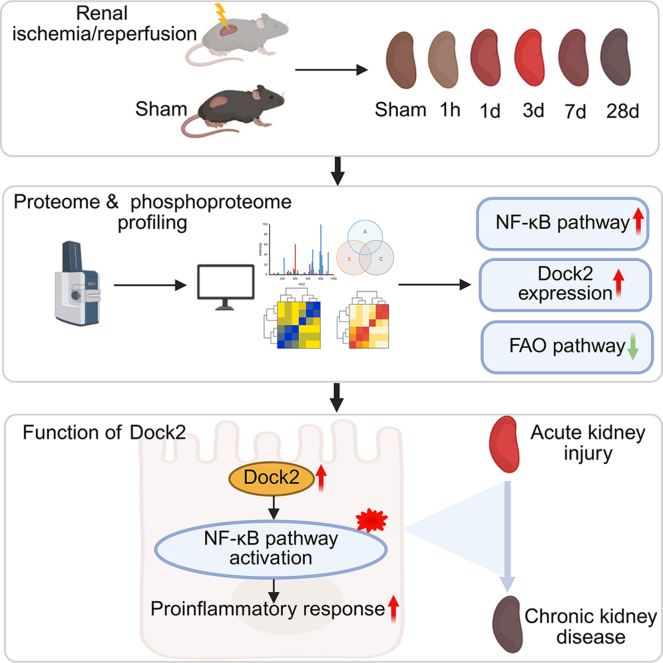

## In Brief

This 4D label-free proteomic and phosphoproteomic profiling systematically identified dysregulated proteins, phosphoproteins, and signaling pathways in AKI-to-CKD transition post-IRI. The enrichment and sustained activation of NF-κB signaling and impaired fatty acid β-oxidation (FAO) were revealed. Genetic and pharmacological targeting demonstrated Dock2 as a key mediator of proinflammatory responses in renal tubule cells via the IKKβ/NF-κB pathway. These findings nominate Dock2 as a promising therapeutic target to mitigate AKI-to-CKD progression post-IRI.

Acute kidney injury (AKI) is a group of clinical syndromes characterized by acute renal dysfunction, which is a common complication in hospitalized patients and independently associated with increased morbidity and mortality (1). Many studies have shown that survivors of AKI have a significantly increased risk of suffering chronic kidney disease (CKD), which results in another enormous burden on health systems (1, 2). Renal ischemia-reperfusion injury (IRI), which occurs in kidney transplantation, hemorrhage, cardiac arrest, or shock, represents a major cause of AKI (3). In spite of extensive research of AKI, the clinical outcome for those suffering AKI has not been improved dramatically (4). The precise mechanisms underlying the progression from AKI to CKD after IRI remain unclear.

Previous studies have suggested that the pathogenesis of the AKI-to-CKD transition following IRI involves tubular epithelial cell (TEC) damage, inflammatory responses, lipid metabolism disorders, cell cycle arrest, vascular endothelial injury, and renal interstitial fibrosis (5, 6). Zheng *et al*. and Rossi *et al*. reported that ischemia/reperfusion (I/R)-injured TECs secrete proinflammatory mediators (e.g. interleukin-6 (IL-6), tumor necrosis factor-α (TNF-α), and monocyte chemoattractant protein-1 (MCP-1)), triggering an inflammatory response that promotes the AKI-to-CKD progression (7, 8). Yang *et al*. demonstrated that after severe insults such as IRI, tubular G2/M arrest occurs, leading to the production of profibrotic factors (e.g. transforming growth factor beta 1 (TGF-β1) and connective tissue growth factor (CTGF)) by TECs, thereby driving the AKI-to-CKD transition (9). Furthermore, Zhu *et al*. suggested that IRI induces lipid metabolism disorders in TECs, resulting in lipid accumulation, inflammation, and fibrosis, which collectively contribute to the transition from AKI to CKD (6). Perry *et al*. demonstrated that the vascular endothelium plays a critical role in both alleviating IRI-induced AKI and promoting renal recovery (10). In addition, Nakamura *et al*. reported that fibroblasts and pericytes can differentiate into myofibroblasts, which produce extracellular matrix components (e.g. fibronectin and collagen), thereby accelerating CKD progression after AKI (11). However, the temporal global AKI-to-CKD transition and phosphoproteomic alterations underlying AKI-to-CKD progression following IRI remain largely unexplored.

Using global proteomic and phosphoproteomic analyses in a murine unilateral IRI model at five time points post injury, we characterized molecular signatures and identified key pathways involved in AKI-to-CKD transition. These analyses consistently revealed the enrichment and sustained activation of NF-κB signaling, as well as markedly impaired fatty acid β-oxidation (FAO). Notably, the dedicator of cytokinesis (Dock) protein family was identified, with Dock2 emerging as a key candidate. Genetic knockdown and pharmacological inhibition experiments suggested the critical role of Dock2 in mediating the inflammatory response in renal IRI. Our findings nominate Dock2 as a therapeutic target for mitigating the progression from AKI to CKD following IRI.

## Experimental Procedures

### Animals

All mouse experiments were approved by the Animal Experiment Ethics Committee of the Affiliated Hospital of Guangdong Medical University (Approval No. 202209–0052). Male C57BL/6J mice (6–8 weeks old, 20–24 g) were purchased from SPF (Beijing) Biotechnology Co, Ltd. Mice were housed in a standard environment under a 12-h light/dark cycle, with controlled temperature (22–25 °C) and humidity (40–60%). Water and forage were freely available.

### Animal Experimental Protocol

In this study, the renal IRI was performed based on the model described in the previous report (12). Briefly, all mice were anesthetized by intraperitoneal (i.p.) injection of sodium pentobarbital. To maintain the body temperature at about 37 °C, the mice were placed on heating devices. The left renal pedicle was exposed though a 2 to 3 cm midline laparotomy and then clipped for 35 min by using the vascular clamp. After removing the clamp, the kidney returned to normal color from dark purple. Sham surgeries were carried out with exposure of left kidneys but without the induction of ischemia. To assess renal function, the right kidney was removed 24 h prior to euthanizing the mice. Mice were sacrificed at 1 h, 1 day, 3 days, 7 days, and 28 days after reperfusion. Renal tissues and blood samples were harvested for the purpose of various analyses.

To assess the effect of 4-[3′-(2″-chlorophenyl)-2′-propen-1′-ylidene]-1-phenyl-3,5-pyrazolidinedione (CPYPP, Dock2 inhibitor) (13) on the renal IRI, mice were randomized into five groups (n = 4): the Sham group, IRI 3 days + vehicle group, IRI 3 days + CPYPP group, IRI 28 days + vehicle group, and IRI 28 days + CPYPP group. IRI was induced as described above. The mice in the IRI 3 days + CPYPP group received CPYPP (250 mg/kg body weight; catalog no. HY-110100, MedChem Express; dissolved according to the manufacturer’s instructions), while those in the IRI 3 days + vehicle group received an equivalent volume of vehicle, both via i.p. injection 30 min after I/R; all mice were sacrificed at 3 days after I/R. The mice in the IRI 28 days + CPYPP group received CPYPP (250 mg/kg per injection) via i.p. injection at 30 min, 7 days, 14 days, and 21 days after I/R, while the IRI 28 days + vehicle group received an equivalent volume of vehicle at the same time points; all mice were sacrificed at 28 days after I/R.

To specifically determine the contribution of NF-κB to the sustained upregulation of IL-6 post-IRI, we inhibited NF-κB p65 nuclear translocation using JSH-23 (14). Mice were randomly assigned to five groups (n = 4 per group): Sham, IRI 1 day + vehicle, IRI 1 day + JSH-23, IRI 28 days + vehicle, and IRI 28 days + JSH-23. IRI was induced as described above, and the right kidney was removed 24 h prior to euthanasia. Mice in the IRI 1 day + JSH-23 group received JSH-23 (20 mg/kg per injection; MedChem Express, HY-13982, dissolved according to the manufacturer’s instructions) via i.p. injection at 12 h before and after I/R, while the corresponding vehicle group received an equivalent volume of vehicle; all mice in these groups were sacrificed at 1 day after I/R. For the IRI 28 days + JSH-23 group, JSH-23 was administered via i.p. injection at 20 mg/kg per injection at 12 h before and after I/R, followed by additional injections (10 mg/kg per injection) at 3, 5, 7, 9, 11, 13, 15, 17, 19, 21, 23, 25, and 27 days after I/R; the IRI 28 days + vehicle group received vehicle at the same time points and volumes, and all mice were sacrificed at 28 days after I/R. To analyze NF-κB activation and IL-6 expression, nuclear and cytoplasmic proteins were extracted from renal tissues using the NE-PER Nuclear and Cytoplasmic Extraction Reagents (Thermo Fisher Scientific, catalog no. 78835) according to the manufacturer’s instructions for subsequent western blot analysis.

### Renal Function Assessment

Serum was obtained by centrifugation of murine whole blood at 3000*g* for 10 min at 4 °C, followed by supernatant aspiration. Serum creatinine (Scr) concentrations were determined with a standardized creatinine detection kit (Jiancheng Bio, C011-2-1) in accordance with the manufacturer’s guidelines.

### Pathology Staining

Mouse tissue preparation—Kidney tissues collected at designated time points were either fixed in 4% paraformaldehyde for 24 h followed by paraffin embedding or embedded directly in Tissue OCT-Freeze Medium (SAKURA).

H&E staining and Masson’s staining—The paraffin-embedded kidney sections (3 μm) were stained with H&E (Beyotime, C0105M) and Masson's trichrome (Applygen, B1130) according to the manufacturers' standard protocols. The pathological changes were scored by a pathologist who was required to be blind to the grouping. Through H&E staining, the degree of tubular injury, defined as dilated, necrotic, atrophied tubules, brush border loss, as well as tubular casts, was scored with the below criteria: 0, no pathological evidence of tubular injury; 1, <25% tubules involved; 2, 25%–50% tubules involved; 3, 51%–75% tubules involved; 4, >75% tubules involved. Masson’s staining was used to assess the tubulointerstitial fibrosis, the degree of which was scored according to the following standard: 0, no pathological evidence of tubulointerstitial fibrosis; 1, <25% tubulointerstitium involved; 2, 25%–50% tubulointerstitium involved; 3, >50% tubulointerstitium involved. So as to obtain more accurate assessment, the average of 10 random high-power fields (400×) was set as the score for every kidney sample.

Immunohistochemistry—The kidney sections (3 μm), embedded in paraffin, underwent dewaxing as previously described (15). Antigen retrieval was achieved by boiling in retrieval solution (Beyotime, P0083) for 10 min at 100 °C. Following blocking, the sections were incubated overnight at 4 °C with an anti-Havcr1/Kim1 primary antibody (R&D Systems, cat. AF1817, dilution 1:200), then treated with horseradish peroxidase (HRP)-conjugated secondary antibody at room temperature (RT) for 1 h, and finally developed with 3,3′-diaminobenzidine (DAB) substrate (ZSGB, ZLI-9017). Nuclear staining was performed using hematoxylin. Immunohistochemical images were acquired using an Olympus IX81 light microscope.

### Proteomic and Phosphoproteomic Analyses

Sample preparation—Protein lysates were extracted from kidney tissues by lysis in buffer (100 mM DTT, 4% SDS, 100 mM Tris–HCl pH 8.0), with subsequent 3-min boiling and 2-min ultrasonication. Undissolved debris was removed by centrifugation at 16,000*g* for 20 min at 4 °C. Protein concentrations in supernatants were analyzed by the bicinchoninic acid assay (Bio-Rad). Proteins were digested using the filter-aided sample preparation (FASP) method as described by Wiśniewski *et al* (16). Briefly, after reduction (100 mM DTT, 100 °C, 5 min) and alkylation (50 mM iodoacetamide, RT, 30 min, dark), proteins were digested with trypsin (Promega) at 37 °C for 16 to 18 h. The peptides were collected by centrifugation (12,000*g*, 10 min) and desalted using spin columns (Thermo Fisher Scientific). For phosphoproteomic analysis, digested peptides underwent Fe-NTA phosphopeptide enrichment (Thermo Fisher Scientific) according to the manufacturer's instructions.

Liquid chromatography-tandem mass spectrometry (LC-MS/MS) analysis—LC-MS/MS analyses were performed by Bioprofile Co, Ltd using a timsTOF mass spectrometer (Bruker) coupled to an Easy nLC 1200 system (Thermo Fisher Scientific). Peptides in buffer A (0.1% formic acid) were separated on an IonOpticks column (250 mm × 75 μm, 1.6 μm) with buffer B (80% acetonitrile, 0.1% formic acid) at 300 nl/min, using a gradient: 2 to 22% B (45 min), 22 to 35% B (5 min), 35 to 80% B (5 min), 80% B (5 min). Mass spectrometry (MS) analysis was conducted in data-dependent acquisition mode with parallel accumulation-serial fragmentation (PASEF) enabled. Key parameters: m/z range 100 to 1700, ion mobility 0.6 to 1.6 V s/cm^2^, ramp time 100 ms, accumulation time 2 ms, quadrupole isolation width (2 m/z for m/z < 700–3 m/z for m/z > 700), and 24 s dynamic exclusion. Each 1.1 s trapped ion mobility spectrometry (TIMS) cycle comprised 1 full MS scan and 10 PASEF MS/MS scans.

Database search—For protein identification, the MS data were analyzed using MSFragger (v3.4) and searched against the *Mus musculus* database from UniProt (release 2022–12, containing 88,040 protein sequences) (https://www.uniprot.org/). Proteomic and phosphoproteomic analyses were performed with the following parameters: tryptic digestion (maximum of two missed cleavages allowed), 20 ppm mass tolerance for both precursors and fragments, variable modifications (methionine oxidation and N-terminal acetylation), and fixed modification (carbamidomethylation of cysteine). For phosphoproteomic analysis, phosphorylation of tyrosine, serine, and threonine was included as an additional variable modification during database searching. The false discovery rate was set to 1% for peptide-spectrum matches, protein, and phosphosite identifications. In addition, a best localized probability score cutoff of >0.75 was applied to ensure high-confidence phosphorylation site localization.

Quantitative data processing—Relative quantification of proteins and phosphorylated peptides was performed using the label-free quantification module within the MSFragger platform. The match-between-runs feature was enabled. The protein-level label-free quantification intensities were calculated from unique and razor peptides. Median normalization was applied by dividing the intensity of each protein or phosphopeptide in a sample by the median intensity of the entities in that sample.

Statistical and bioinformatic analysis—In this study, analysis of bioinformatic data was performed with Perseus software. Differences between two groups were assessed using Student’s *t* test. For multiple groups, statistical differences were analyzed using one-way ANOVA. Statistical significance was defined as *p* < 0.05. For both proteomic and phosphoproteomic analyses, significantly differential expressions were identified using the following cutoff criteria: *p* < 0.05 with fold change >1.5 or < −1.5 for proteins, and fold change >2 or < −2 for phosphorylation sites. Gene Ontology (GO) enrichment analysis, Mfuzz analysis (fuzzy C-means clustering), Venn diagram construction, and heatmap visualization were performed using the bioinformatics online platform (17) (https://www.bioinformatics.com.cn). Chord diagrams were generated using Origin 2024. The proteins from the enriched GO biological process (BP) terms and the overlapping differentially expressed proteins (DEPs) were analyzed for protein-protein interactions (PPIs) using the STRING database (https://string-db.org/). The PPI networks were visualized with Cytoscape software.

### Cell Culture

The HK-2 human proximal tubular epithelial cell line was obtained from the American Type Culture Collection (ATCC). Cells were maintained in Dulbecco's modified Eagle's medium (DMEM)/Ham's F-12 medium (Gibco) containing 10% fetal bovine serum (Gibco) and 1% penicillin–streptomycin antibiotic mixture, and incubated at 37 °C in a humidified 5% CO_2_ atmosphere (Thermo Fisher Scientific incubator).

### *In Vitro* Hypoxia/Reoxygenation Model

The hypoxia/reoxygenation (H/R) model was established with modifications based on previously published protocols (18). Briefly, cellular hypoxia was induced by 24-h incubation in glucose- and serum-free medium under 1% O_2_/5% CO_2_/94% N_2_ atmosphere at 37 °C (ESCO anaerobic chamber). Following hypoxia, cells were reoxygenated for 12 h in complete medium under normoxic conditions. Control cells were maintained in complete medium under normoxic conditions.

### RNA Interference

Cells at 50 to 70% confluence were transiently transfected with siRNAs using Lipofectamine 3000 (Invitrogen) following the manufacturer's protocol. The sequences of Dock2-si1 were as follows: sense 5′-GGUCAUCUCUGGAAUCUAATT-3′, and antisense 5′-UUAGAUUCCAGAGAUGACCTT-3′. The sequences of Dock2-si2 were as follows: sense 5′-CCACCAUACCAAUCUUCUUTT-3′, and antisense 5′-AAGAAGAUUGGUAUGGUGGTT-3′. The sequences of negative control siRNA (Con-si) were: sense 5′-UUCUCCGAACGUGUCACGUTT-3′, and antisense 5′-ACGUGACACGUUCGGAGAATT-3′. All siRNAs were designed and synthesized by GenePharma.

### Reverse Transcription Quantitative PCR (RT-qPCR)

Total RNA from cells was extracted using TRIzol reagent (Invitrogen) according to the manufacturer's instructions. After assessing RNA quality, complementary DNA was synthesized using a PrimeScript RT reagent kit with gDNA Eraser (Takara), followed by real-time PCR with Genious SYBR Green Fast qPCR Mix (ABclonal, RK21204), both performed according to the manufacturers’ protocols. β-actin served as an inner control. All primers for the selected genes were as follows: for IL-6(h), forward 5′-CCTGAACCTTCCAAAGATGGC-3′, and reverse 5′-TTCACCAGGCAAGTCTCCTCA-3′; for MCP-1(h), forward 5′-CAGCCAGATGCAATCAATGCC-3′, and reverse 5′-TGGAATCCTGAACCCACTTCT-3′; for TNF-α(h), forward 5′-CCTCTCTCTAATCAGCCCTCTG-3′, and reverse 5′-GAGGACCTGGGAGTAGATGAG-3′; for β-actin(h), forward 5′-CATTCCAAATATGAGATGCGTTGT-3′, and reverse 5′-GCATTACATAATTTACACGAAAGC-3′.

### Immunofluorescence

Immunofluorescent staining was carried out as described previously (19). In brief, after fixation with 4% paraformaldehyde, permeabilization with 0.5% Triton X-100, and blocking with 1% bovine serum albumin, frozen kidney sections or HK-2 cells were incubated overnight at 4 °C with primary antibodies against F4/80 (Bio-Rad, MCA497G, dilution 1:200), Dock2 (Millipore, cat. 09–454, dilution 1:100), Havcr1/Kim1 (R&D Systems, cat. AF1817, dilution 1:200) and NF-κB p65 (Abcam, cat. ab32536, dilution 1:100). The sections/cells were then incubated with Alexa Fluor-conjugated secondary antibodies at RT for 1 h (protected from light). Nuclear staining was achieved using 4′,6-diamidino-2-phenylindole (DAPI). Images were captured by the confocal microscope (Olympus, FV3000). The F4/80-positive area in the renal cortex was quantified using ImageJ software. For each kidney sample, ten random high-power fields were analyzed, and the average percentage of positive area was calculated as the quantitative score.

### Western Blot Assay

In our experiment, the western blotting was performed as reported previously (20). Briefly, proteins were extracted by lysing tissues or cells in RIPA buffer (Beyotime) supplemented with protease inhibitor cocktail (Beyotime) and phosphatase inhibitor (Applygen), and then quantified by bicinchoninic acid assay (Thermo Fisher Scientific). After denaturation, the proteins were separated by SDS-PAGE and then transferred onto polyvinylidene difluoride membranes (Millipore). The membranes were incubated with primary antibodies at 4 °C overnight after being blocked in 5% bovine serum albumin. We chose the following antibodies in this study: anti-Havcr1/Kim1 (R&D Systems, cat. AF1817, dilution 1:1000); anti-IL-6 (Cell Signaling Technology, cat. 12912S, dilution 1:1000); anti-MCP-1 (Cell Signaling Technology, cat. 2029S, dilution 1:1000); anti-TNF-α (Cell Signaling Technology, cat. 11948S, dilution 1:1000); anti-Dock2 (Millipore, cat. 09–454, dilution 1:500); anti-IKKβ (Starter, cat. S0B1387, dilution 1:1000); anti-phospho-IKKα/β (Ser176/180) (Cell Signaling Technology, cat. 2697S, dilution 1:1000); anti-Fibronectin (Sigma-Aldrich, cat. SAB4500974, dilution 1:1000); anti-α-SMA (Sigma-Aldrich, cat. A5228, dilution 1: 1000); anti-GAPDH (Absin, cat. abs830030, 1:2000). Afterward, secondary antibodies were used to incubate the membranes at RT for 1 h. Finally, blots were visualized using Clarity Western ECL Substrate (Bio-Rad) and imaged with an Azure Biosystems C500 system.

### Experimental Design and Statistical Rationale

This study employed a longitudinal design using a murine unilateral IRI model sampled at critical time points post-IRI (1 h, 1 day, 3 days, 7 days, and 28 days). Sham-operated mice served as controls. Kidney tissues from three biologically independent mice per group underwent integrated 4D label-free proteomic and phosphoproteomic analysis to globally identify dysregulated proteins, phosphoproteins, and pathways. Key omics findings included Dock2 upregulation, which was confirmed by western blot and immunofluorescence. Functional validation of Dock2 was performed through *in vitro* siRNA-mediated knockdown in TECs and *in vivo* pharmacological inhibition using CPYPP, both specifically targeting Dock2. All grouped data were presented as mean ± SEM. Box plots displayed expression data as medians with interquartile ranges. Differences between two groups were assessed using Student’s *t* test. For multiple groups, statistical differences were analyzed using one-way ANOVA followed by Fisher's least significant difference (LSD) post hoc test. Statistical significance was defined as *p* < 0.05.

## Results

### Time-dependent Changes in TECs Injury, Inflammatory Infiltration, and Fibrotic Remodeling Following Renal IRI

**Fig. 1:**
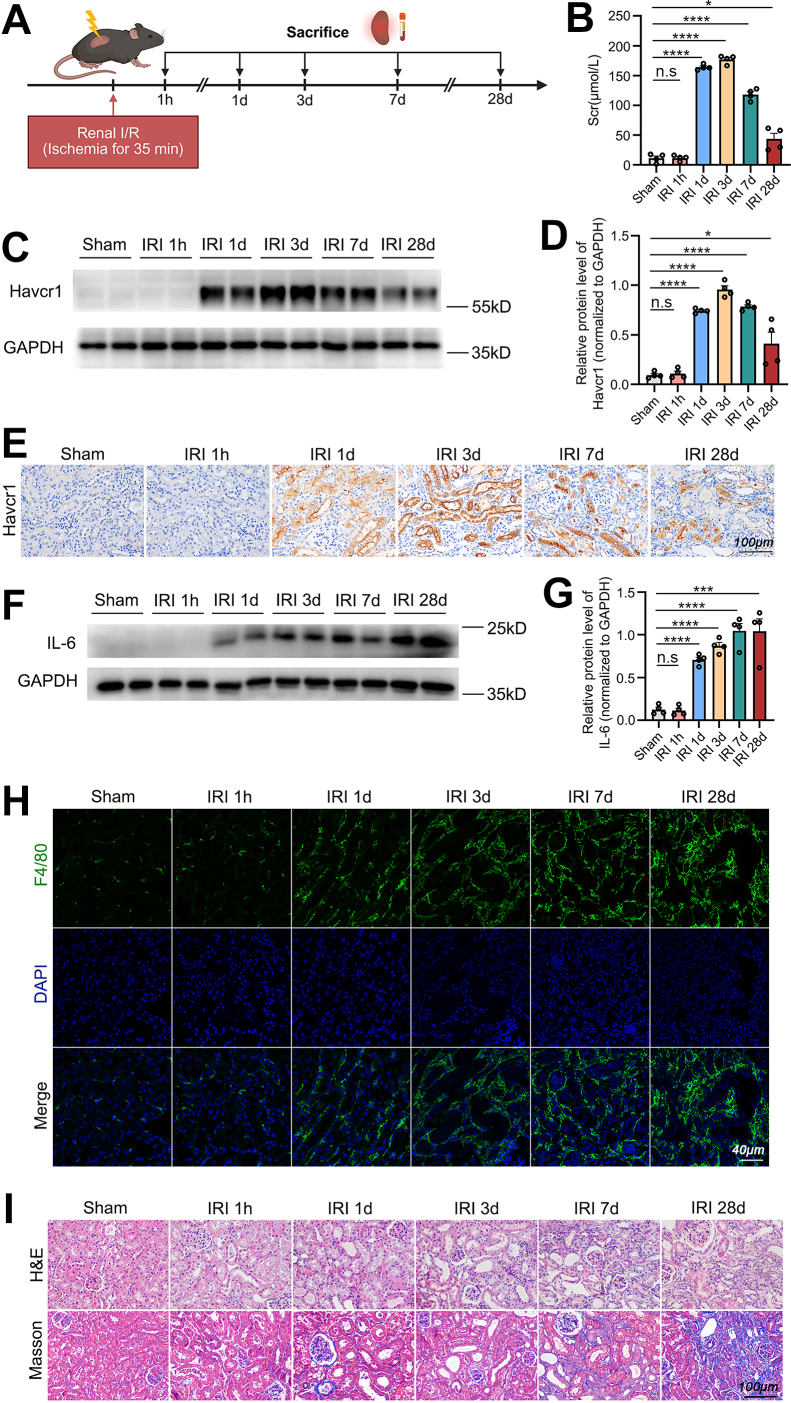
**Ischemia-reperfusion injury elicits time-dependent changes of TECs injury, inflammatory infiltration, and interstitial fibrotic remodeling.***A*, schematic illustration of the experimental protocol for inducing renal IRI in a mouse model. *B*, levels of Scr from sham-operated mice and mice sacrificed at 1 h, 1 day, 3 days, 7 days, 28 days after I/R. *C* and *D*, protein expression profiling by western blot (*C*) and subsequent statistical analysis (*D*) of Havcr1/Kim1 in renal cortical samples. *E*, immunohistochemical detection of Havcr1/Kim1 expression in renal cortical sections from sham and I/R-treated mice. The scale bar represents 100 μm. *F* and *G*, protein expression profiling by western blot (*F*) and subsequent statistical analysis (*G*) of IL-6 in renal cortical samples. *H*, immunofluorescent staining for the macrophage marker F4/80 in renal cortical sections. The scale bar represents 40 μm. *I*, histopathological assessment of renal tissue architecture (H&E) and collagen deposition (Masson’s trichrome) in experimental groups. The scale bar represents 100 μm. Data are presented as mean ± SEM. n.s, not significant; ∗*p* < 0.05; ∗∗∗*p* < 0.001; ∗∗∗∗*p* < 0.0001. Havcr, hepatitis A virus cellular receptor 1; IL-6, interleukin-6; IRI, ischemia-reperfusion injury; Kim1, kidney injury molecule-1; Scr, serum creatinine; TEC, tubular epithelial cell.

With the aim of identifying the key pathways and molecular mechanisms underlying the AKI-to-CKD transition, our study established a mouse model of renal IRI. To obtain dynamic changes in renal pathology, mice were sacrificed at five different time points after IRI: 1 h, 1 day, 3 days, 7 days, and 28 days (Fig. 1*A*). Mouse blood and renal tissues were then collected for further analysis.

As shown in Figure 1*B*, Scr levels were significantly elevated at 1 day and 3 days post-IRI versus sham controls. Although Scr levels declined at 7 days and 28 days post-IRI relative to the 3-day time point, they remained elevated above baseline levels. Western blot analysis showed that hepatitis A virus cellular receptor 1 (Havcr1), a tubular injury biomarker also known as kidney injury molecule-1 (Kim1), followed a trend similar to that of Scr. Havcr1/Kim1 expression was markedly upregulated at 1 day and 3 days post-IRI, followed by a decline at 7 days and 28 days compared to the 3-day peak (Fig. 1, *C* and *D*). Consistent with the western blot findings, immunohistochemical analysis revealed a parallel temporal pattern of Havcr1/Kim1 expression (Fig. 1*E*). It is generally accepted that inflammatory response is an important pathological process in the development and progression of AKI (21). We examined an inflammatory mediator, IL-6 (22), by western blot. As expected, IL-6 expression was significantly upregulated at 1 day, 3 days, 7 days, and 28 days post-IRI compared to sham controls (Fig. 1, *F* and *G*). Consistent with this result, immunofluorescent analysis confirmed a time-dependent increase in F4/80^+^ macrophage infiltration from 1 day to 28 days post-IRI (Fig. 1*H*), demonstrating that IRI induces a robust inflammatory response.

H&E staining of kidney sections revealed distinct pathological features across IRI phases: brush border sloughing in the hyperacute phase (1 h); tubular dilation, brush border loss, cast formation, and increased interstitial cellularity in acute phases (1 day and 3 days); and tubular atrophy, interstitial expansion, and further increased interstitial cellularity in progressive stages (7 days and 28 days) (Fig. 1*I*). Further, Masson’s staining showed that in the progressive stages (7 days and 28 days) of IRI, the kidneys presented focal tubulointerstitial expansion and interstitial fibrosis (Fig. 1*I*).

Collectively, the above results indicated that IRI results in progressive alteration of renal tissues, which satisfies our aim to model the AKI-CKD transition.

### Overlapping Differentially Expressed Proteins in Renal Proteome Post-IRI and Their Functional Enrichment

**Fig. 2:**
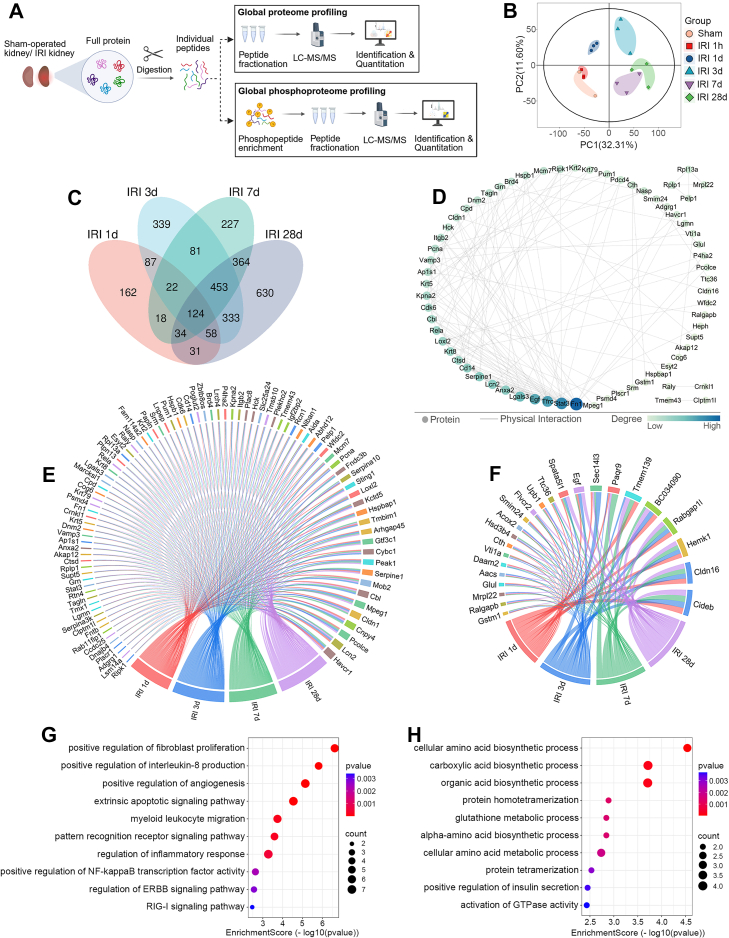
**Proteomic analysis of renal tissues identifies overlapping differentially expressed proteins at 1, 3, 7, and 28 days post-IRI versus sham controls, followed by functional enrichment analysis.***A*, concise experimental procedure of the study. *B*, principal component analysis to show within-group consistency and between-group difference. *C*, venn diagram illustrates the overlap of DEPs among comparisons: IRI 1 day versus sham, IRI 3 days vs. sham, IRI 7 days versus sham, and IRI 28 days versus sham. *D*, PPI network of the overlapping DEPs across all four post-IRI time points in (*C*). The *blue*-to-*green* color gradient and circle size represent the degree of each protein in the PPI network. *E* and *F*, chord diagram showing the correspondence between post-IRI time points and the overlapping upregulated (*E*) or downregulated (*F*) proteins across all post-IRI time points in (*C*). The length of each segment is quantitatively determined by the summation of |log2 FC| of each protein. *G* and *H*, functional enrichment analysis of GO biological processes (BP) for overlapping upregulated (*G*) or downregulated (*H*) proteins across all post-IRI time points in (*C*). DEP, differentially expressed protein; FC, fold change; GO, Gene Ontology; IRI, ischemia-reperfusion injury; PPI, protein-protein interaction.

In order to obtain an unbiased renal proteome profile of sham controls and IRI mice at 1 h, 1 day, 3 days, 7 days, and 28 days post injury, LC-MS/MS analysis was performed following a 4D label-free quantitative strategy, as showed in Figure 2*A*. Proteomic profiling identified 8051 proteins (Supplemental Table S1), of which 7541 qualified for differential expression analysis (Supplemental Table S2). To assess the data quality of proteome, principal component analysis demonstrated that our data segregated clearly into six groupings (Fig. 2*B*). As expected, the sham and 1 h post-IRI groups clustered in proximity, while distinct groupings emerged for the 1 day, 3 days, 7 days, and 28 days time points (Fig. 2*B*).

Subsequently, post-IRI protein expression (1 day–28 days) was quantitatively compared with sham controls. As a result, 536 proteins (137 downregulated and 399 upregulated) in the IRI 1 day group versus sham group, 1497 proteins (377 downregulated and 1120 upregulated) in the IRI 3 days group versus sham group, 1323 proteins (400 downregulated and 923 upregulated) in the IRI 7 days group versus sham group, and 2027 proteins (744 downregulated and 1283 upregulated) in the IRI 28 days group versus sham group were identified to be the DEPs (Supplemental Table S3). Furthermore, Venn analysis revealed that 124 DEPs overlapped across these four pairs of comparisons (Fig. 2*C*) (Supplemental Table S4), highlighting their potential as core mediators of IRI progression. PPI network analysis of the overlapping DEPs identified five hub proteins with the highest degree values: Fn1, signal transducer and activator of transcription 3 (Stat3), Tfrc, Egf, and Galectin-3 (Lgals3) (Fig. 2*D*). These hubs are known to regulate critical pathways in IRI progression, including extracellular matrix remodeling (Fn1) (23), inflammatory signaling (Stat3, Lgals3) (24, 25), iron metabolism (Tfrc) (26), and epithelial repair (Egf) (27). To visualize the overlapping DEPs, chord diagrams were constructed to show the correspondence between post-IRI time points and the upregulated (Fig. 2*E*) or downregulated (Fig. 2*F*) proteins. The chord diagram of upregulated proteins (Fig. 2*E*) revealed 91 prominent targets including: Havcr1/Kim1, a well-established AKI biomarker that has also been associated with renal inflammation and fibrosis (28); Lcn2 (NGAL), also an early AKI marker that reflects renal impairment severity and is involved in CKD pathophysiology (29); Cldn1 (Claudin-1), a tight junction protein shown to contribute to inflammation and fibrosis (30), though its role in AKI or CKD remains unclear. The diagram of downregulated proteins (Fig. 2*F*) comprised 24 targets, notably: Cldn16, a key regulator of paracellular calcium transport whose dysfunction correlates with hypercalciuria and nephrocalcinosis (31); Rabgap1l, a TBC/Rab GAP family member regulating selective autophagy (32); cell death-inducing DFFA-like effector B (Cideb), a lipid metabolism regulator whose downregulation is associated with metabolic dysregulation (33), though its role in renal pathophysiology requires further investigation.

The current study performed GO enrichment analysis to examine the BP terms associated with the overlapping DEPs. As shown in Figure 2*G*, GO analysis of the overlapping upregulated proteins highlighted three functional clusters: 1) Inflammatory activation, involving NF-κB signaling (e.g. Rela, Stat3, and Ripk1), IL-8 production (e.g. Cd14, Serpine1 and Rela), and myeloid leukocyte migration (e.g. Itgb2, Lgals3 and Rtn4); 2) Immune recognition and apoptosis, characterized by pattern recognition receptor signaling (e.g. Lsm14a, Pum1 and Cd14) and extrinsic apoptosis pathways (e.g. Lcn2, Krt8 and Tmbim1); 3) Fibrotic remodeling and repair, driven by fibroblast proliferation (e.g. Fn1, Anxa2 and Cdk6) and angiogenesis (e.g. Hspb1, Grn, and Rtn4) (Supplemental Table S5). Notably, NF-κB signaling showed functional crosstalk with the other GO BP mentioned above (34, 35, 36), suggesting its important role in mediating the AKI-to-CKD transition through coordinated inflammation and fibrosis. In contrast, the overlapping downregulated proteins (e.g. Cth, Glul, and Upb1) were significantly enriched in BP terms, such as biosynthetic processes of amino acid, carboxylic acid and organic acid (Fig. 2*H*) (Supplemental Table S6). Previous studies indicated that these metabolic pathways, particularly involving branched-chain amino acids, glutamine, arginine, and tryptophan, are crucial for renal homeostasis and may influence AKI progression (37, 38).

### Proteomic Temporal Clustering Identifies protein Modules Linked to renal Injury in Post-IRI Kidneys

**Fig. 3:**
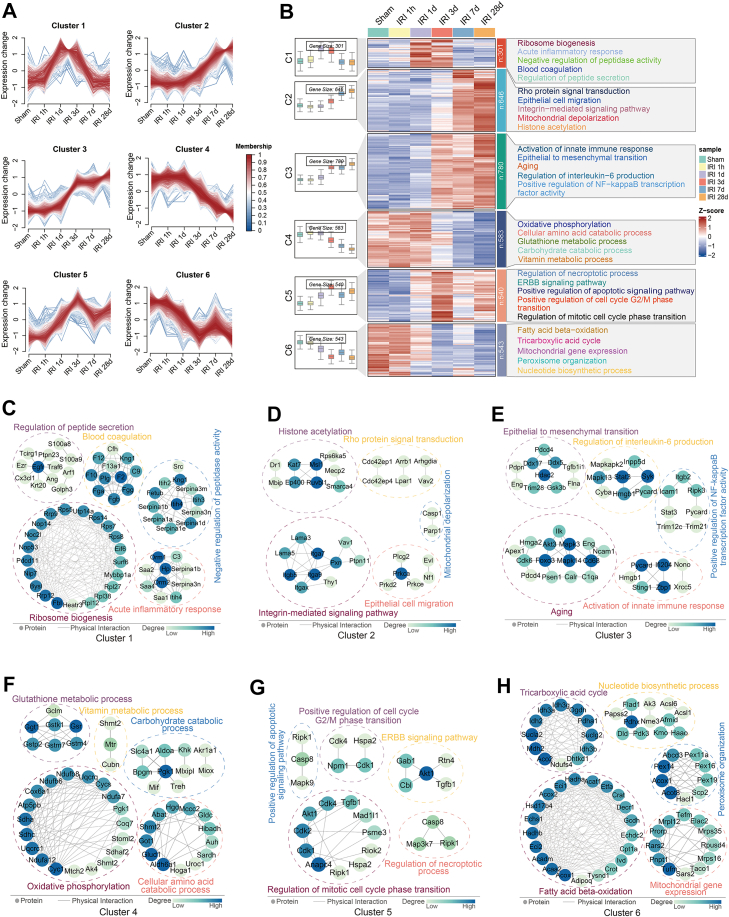
**Proteomic temporal clustering reveals dynamic protein modules annotated with GO terms in post-IRI kidneys.***A*, proteins were clustered temporally based on their post-IRI expression dynamics using Mfuzz analysis. *B*, the heatmap highlights six protein clusters exhibiting distinct abundance dynamics. Numerical labels in the *left* boxes represent protein counts for each cluster. The *right* panel displays enriched GO BP terms for each protein cluster. *C*–*H*, PPI networks of proteins from the enriched GO BP terms in clusters 1 to 6 of (*B*) (*C*: Cluster 1, *D*: Cluster 2, *E*: Cluster 3, *F*: Cluster 4, *G*: Cluster 5, *H*: Cluster 6). The *blue*-to-*green* color gradient represents the degree of each protein in the PPI network. BP, biological process; GO, Gene Ontology; IRI, ischemia-reperfusion injury; PPI, protein-protein interaction.

Our study investigated the dynamic protein expression profiles during the progression of IRI. The fuzzy C-means (FCM) clustering algorithm, a classical soft clustering method in data mining, was employed to analyze these patterns. Using the Mfuzz package (an FCM-based tool optimized for biological data), we identified six distinct time-dependent protein expression clusters (Fig. 3*A*). GO enrichment analysis revealed distinct BP associated with each cluster. These findings were synthesized in a functional heatmap (Fig. 3*B*), integrating temporal protein expression dynamics of the six clusters (from Fig. 3*A*), and significantly enriched GO BP terms (Supplemental Table S7). For proteins annotated to the enriched GO BP terms in each cluster, PPI networks were constructed using the STRING database (Fig. 3*C*–*H*).

Proteins in cluster 1 exhibited a characteristic acute-phase response pattern, showing upregulation at 1 day and 3 days post-IRI, with enriched BP terms such as acute inflammatory response, ribosome biogenesis, and negative regulation of peptidase activity (Fig. 3*B*). Of note, the proteins annotated to the acute inflammatory response include Orm1, Orm2, serum amyloid A 1 (Saa1), and Saa2 (Fig. 3*C*). Orosomucoid 1 (Orm1) has been identified as a urinary biomarker for early lupus nephritis (39), but its role in AKI following IRI remains unclear. Previous studies have shown that serum levels of Saa1 and Saa2 significantly increase after trauma, infection, or other stimuli (40). These findings suggest that Saa1 and Saa2 may serve as potential predictors for AKI occurrence. Cluster 2 proteins showed gradual upregulation at 3 days, 7 days, and 28 days post-IRI (Fig. 3*B*), and were enriched in Rho signaling (Cdc42ep1, Cdc42ep4, Arrb1, and so on), epithelial migration (Prkca, Prkd2, Prkce, and so on), and mitochondrial depolarization (Casp1 and Parp1) (Fig. 3*D*). Cluster 3 proteins exhibited early induction at 1 day and a sustained upregulation pattern at 3 days, 7 days, and 28 days post-IRI, enriched in innate immune response, NF-κB signaling, IL-6 production, epithelial to mesenchymal transition and aging (Fig. 3*B*). Our analysis revealed that Itgb2, Icam1, Ripk3, and Stat3 were enriched in NF-κB signaling pathway (Fig. 3*E*). It is noteworthy that integrin beta 2 (Itgb2) has been found to mediate severe acute pancreatitis-related acute lung injury (41). However, the role of Itgb2 in inflammation during AKI or AKI-to-CKD is still unknown. Proteins in cluster 4 showed a continuous suppression pattern at 3 days, 7 days, and 28 days post-IRI (Fig. 3*B*). This cluster was significantly enriched for BP terms including oxidative phosphorylation, amino acid catabolism, glutathione metabolism, and carbohydrate catabolism (Fig. 3*F*). Cluster 5 displayed early upregulation at 1 day and 3 days post-IRI, followed by minor fluctuations at 7 days and 28 days post-IRI, with significant enrichment in BP terms including regulation of necroptotic process, ERBB signaling pathway, apoptotic signaling pathway, and positive regulation of cell cycle G2/M phase transition (Fig. 3*B*). Specifically, proteins involved in the regulation of necroptosis, including Map3k7, Casp8, and Ripk1, were detected (Fig. 3*G*). Accumulating evidence indicates that necroptosis plays an important role in the pathogenesis of various types of AKI (42). However, the regulatory role of Map3k7 in necroptosis during AKI pathogenesis is unknown. Cluster 6 exhibited an early decline at 1 day and 3 days post-IRI with subsequent minor fluctuations at 7 days and 28 days post-IRI, and showed significant enrichment of proteins involved in FAO, tricarboxylic acid cycle (TCA cycle), and mitochondrial gene expression (Fig. 3*B*). Sustained FAO deficiency promotes renal lipotoxicity, inflammation and fibrosis, facilitating AKI-to-CKD transition (6). In cluster 6, the proteins associated with FAO included Acox1, Acaa2, Acadm, and Eci2 (Fig. 3*H*). Therefore, targeting these FAO-related genes may represent a potential therapeutic strategy to prevent AKI-to-CKD progression, warranting further investigation.

Building upon the observed sustained upregulation of IL-6 (Fig. 1, *F* and *G*) and the enrichment of NF-κB signaling in our proteomic analysis (Cluster 3, Fig. 3*B*), we functionally investigated this pathway using JSH-23, an inhibitor of NF-κB p65 nuclear translocation (experimental design in Supplemental Fig. S1, *A* and *B*). Western blot analysis of nuclear extracts showed that JSH-23 treatment effectively suppressed the IRI-induced accumulation of p65 at both 1 day and 28 days post-IRI, reducing it to levels comparable to the sham group (Supplemental Fig. S1, *C* and *D*). Concurrent analysis of cytoplasmic extracts demonstrated that this NF-κB inhibition led to a marked downregulation of IL-6 expression, although its levels did not fully return to the sham baseline (Supplemental Fig. S1, *E* and *F*). These results demonstrate that the sustained upregulation of IL-6 post-IRI is predominantly, but not exclusively, mediated by the NF-κB pathway.

### Dynamic Changes of the Overlapping Differentially Expressed Phosphosites in Renal Tissue Post-IRI

In order to obtain more insights into phosphorylation modification of kidney proteins after IRI, phosphoproteome analysis were performed to the same set of renal samples used for proteome analysis (as shown in Fig. 2*A*). Our study identified a total of 14,502 phosphosites, the best localized probability of which was >0.75, corresponding to 4431 nonredundant phosphoproteins (Supplemental Tables S8 and S9). Of note, 1551 out of the 4431 phosphoproteins were identified exclusively from the phosphoproteomics experiments (Supplemental Fig. S2), implying that phosphopeptide enrichment not only helped to characterize phosphoproteins but also identified low-abundant proteins.

**Fig. 4:**
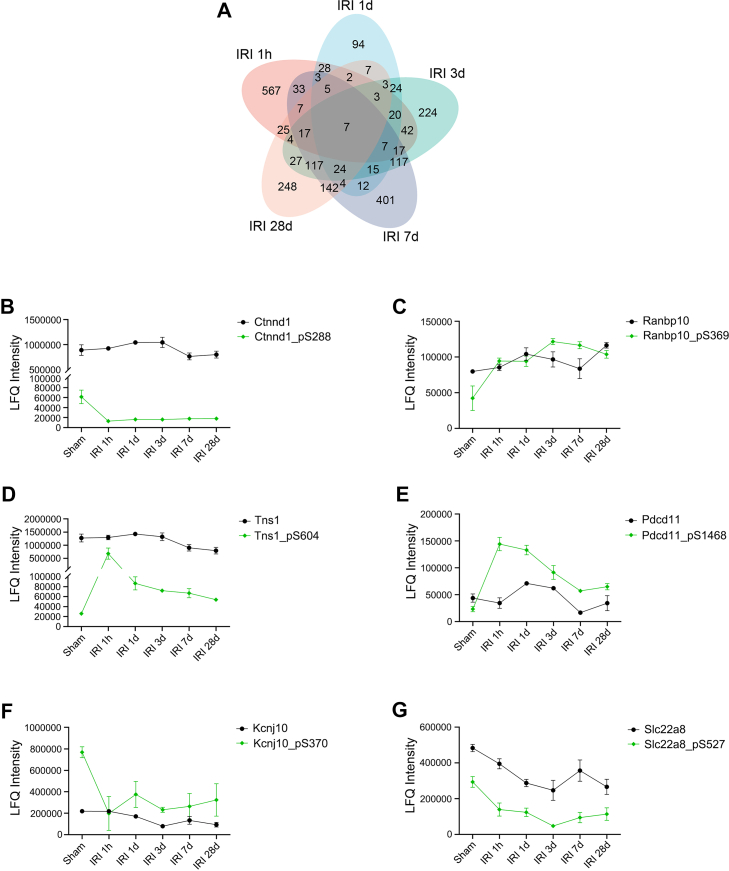
**Dynamic changes of the overlapping differentially expressed phosphosites.***A*, Venn diagram illustrates the overlap of differentially expressed phosphosites in the IRI 1 h group versus sham group, IRI 1 day group versus sham group, IRI 3 days group versus sham group, IRI 7 days group versus sham group, and IRI 28 days group versus. sham group. *B*–*G*, intensity plots of the overlapping differentially expressed phosphosites (*green line*) across all five post-IRI time points in (*A*) and their corresponding total proteins (*black line*). IRI, ischemia-reperfusion injury.

The present study compared the phosphorylation levels of phosphosites at 1 h, 1 day, 3 days, 7 days, and 28 days after IRI with that of sham control, respectively. As a result, differential phosphorylation levels were observed in 787 phosphosites (197 downregulated and 590 upregulated) in the IRI 1 h group versus sham group, 258 phosphosites (136 downregulated and 122 upregulated) in the IRI 1 day group versus sham group, 668 phosphosites (341 downregulated and 327 upregulated) in the IRI 3 days group versus sham group, 928 phosphosites (618 downregulated and 310 upregulated) in the IRI 7 days group versus sham group, and 642 phosphosites (435 downregulated and 207 upregulated) in the IRI 28 days group versus sham group (Supplemental Table S10). Moreover, Venn diagram showed that only seven differentially expressed phosphosites overlapped across these five pairs of comparisons (Fig. 4*A*). These include catenin delta-1 (Ctnnd1, S288), Ran-binding protein 10 (Ranbp10, S369), tensin-1 (Tns1, S604), protein RRP5 homolog (Pdcd11, S1468), ATP-sensitive inward rectifier potassium channel 10 (Kcnj10, S370), organic anion transporter 3 (Slc22a8, S527), and spermatogenesis-associated protein 6 (Spata6, S265) (Supplemental Table S11). Except for Spata6 which was only identified upon phosphopeptide enrichment, the other six of these phosphoproteins were also identified in the proteomic analysis and their temporal expression levels were shown in Figure 4, *B*–*G*. Interestingly, the protein expression levels of both Ctnnd1 and Ranbp10 remained stable among the six groups (ANOVA, *p* > 0.05), whereas their phosphorylated forms changed significantly (ANOVA, *p* < 0.05). The initial analysis of the total phosphorylation signal suggested that phosphorylation of Ctnnd1 at site S288 was downregulated across 1 h, 1 day, 3 days, 7 days, and 28 days after IRI, while phosphorylation of Ranbp10 at site S369 was upregulated across all these time points after IRI. However, to precisely delineate phosphorylation-specific regulation from changes in total protein abundance, we normalized phosphorylation intensities to their corresponding protein levels. This normalized analysis confirmed a statistically significant and sustained dephosphorylation at Ctnnd1 S288, indicating a true posttranslational regulatory event. The detailed temporal profiles of changes in expression (Δ exp) and normalized phosphorylation (Δ phosph, normalized) for the six overlapping phosphoproteins are provided in Supplemental Figure S3. Given these profiles, Ctnnd1 and Ranbp10 are of particular interest due to their stable protein expression alongside significant phosphorylation-specific changes. Ctnnd1, also known as p120-catenin, regulates E-cadherin stability and Rho GTPase activity, as well as interacts with the transcriptional repressor, Kaiso (43). Deletion of Ctnnd1 further leads to inflammation in the lung, skin, pancreas, and intestinal tract (44, 45, 46, 47), as well as tumor initiation or progression in epithelial organs, including skin (45), salivary gland (48), esophagus (49), and liver (50). Previous study has demonstrated that in lung cancer cells, serine 288 phosphorylation in Ctnnd1 enhances the binding with Kaiso and thereby promotes its nuclear export, which is associated with lung cancer progression (43). However, the phosphorylation level of Ctnnd1 at site S288 in response to IRI and its role in AKI or CKD are unknown. Ranbp10 is initially identified as Ran binding proteins and serves as a Ran-GTP exchange factor (GEF) (51). Besides as a GEF, Ranbp10 also acts as a transcriptional regulator (52), and recent studies have shown that Ranbp10 are involved in the cell cycle and tumor progression (53, 54). Phosphorylation of Ranbp10 at site S369 was also identified by previous phosphoproteome analysis of various normal mouse tissues (55). But the phosphorylation level of this site after IRI and its role in kidney is also unknown.

### Phosphoproteomic Temporal Clustering Identifies Phosphorylation Dynamics and Associated Biological Processes in Post-IRI Kidneys

**Fig. 5:**
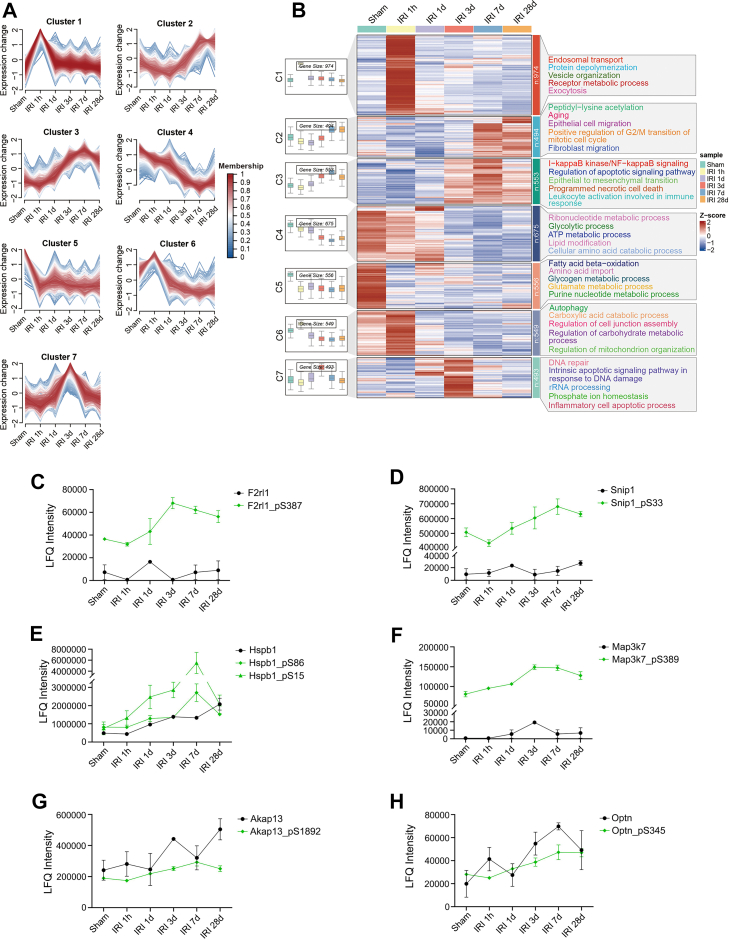
**Phosphoproteomic temporal clustering reveals phosphorylation dynamics with associated GO terms in post-IRI kidneys.***A*, phosphosites were clustered temporally based on their post-IRI expression dynamics using Mfuzz analysis. *B*, the heatmap highlights seven phosphosite clusters exhibiting distinct abundance dynamics. Numerical labels in the *left boxes* represent phosphosite counts for each cluster. The *right panel* displays enriched GO BP terms for each phosphosite cluster. *C*–*H*, intensity plots of the phosphoproteins (*green line*) enriched in the I-kappaB kinase/NF-kappaB signaling pathway in cluster 3 of (*B*) and their corresponding total proteins (*black line*). BP, biological process; GO, Gene Ontology; IRI, ischemia-reperfusion injury.

Through the phosphoproteomic clustering analysis, we identified a total of seven time-dependent expression patterns as well (Fig. 5, *A* and *B*) (Supplemental Table S12). In cluster 1, elevated phosphorylation levels were predominantly observed at 1 h post-IRI for proteins involved in endosomal transport, protein depolymerization, and vesicle organization. In cluster 2, proteins linked to aging, epithelial cell migration, and fibroblast migration showed reduced phosphorylation levels at 1 day post-IRI but became elevated in phosphorylation levels at 7 days and 28 days. In cluster 3, sustained elevated phosphorylation levels were detected at 3 days, 7 days, and 28 days post-IRI for proteins involved in I-kappaB kinase/NF-kappaB signaling, apoptosis, epithelial to mesenchymal transition and programmed necrosis. In clusters 4 and 5, proteins associated with metabolic processes tended to exhibit reduced phosphorylation levels at all time points (1 h, 1 day, 3 days, 7 days and 28 days) post-IRI. This included proteins involved in the ribonucleotide metabolic process (cluster 4) and FAO (cluster 5). Cluster 6 exhibited transiently elevated phosphorylation levels (1 h) then persistent reduced phosphorylation levels (1 day–28 days) for proteins involved in autophagy, regulation of cell junction assembly and mitochondrion organization. In cluster 7, elevated phosphorylation levels were observed at 3 days post-IRI for proteins associated with DNA repair, rRNA processing and phosphate ion homeostasis (Fig. 5*B*).

Among all clusters, cluster 3 was particularly noteworthy. Elevated phosphorylation levels of phosphoproteins in this cluster were observed at 3 days, 7 days, and 28 days post-IRI (Fig. 5*B*). Of particular interest among the enriched GO BP terms in cluster 3 was the I-kappaB kinase/NF-kappaB signaling pathway, given its established role in inflammation, a key driver of AKI-to-CKD progression (56). The phosphoproteins enriched in this signaling pathway include proteinase-activated receptor 2 (F2rl1, S387), smad nuclear-interacting protein 1 (Snip1, S33), heat shock protein beta-1 (Hspb1, S15, and S86), mitogen-activated protein kinase kinase kinase 7 (Map3k7, S389), A-kinase anchor protein 13 (Akap13, S1892), and optineurin (Optn, S345). The temporal expression levels of the above phosphoproteins and their corresponding total proteins were shown in Figure 5, *C*–*H*. Of note, the total protein levels of F2R like trypsin receptor 1 (F2rl1) and Snip1 did not differ significantly among the six groups (ANOVA, p > 0.05), while their phosphorylation forms changed significantly (ANOVA, *p* < 0.05) (Fig. 5, *C* and *D*). F2rl1, also known as proteinase-activated receptor 2 (Par2), is a G protein-coupled receptor (GPCR). F2rl1 activation induces G protein-α-mediated signaling, mobilizing NF-κB signaling (57). Previous studies have demonstrated that F2rl1 deficiency or pharmacological inhibition ameliorates diabetic kidney disease, and diet- or toxin-induced kidney injury in rodent models, by attenuating renal inflammation and fibrosis (58, 59, 60). However, the phosphorylation level of F2rl1 at site S387 in response to IRI and its role in AKI-to-CKD progression have not been determined. Following normalization of phosphorylation to total protein levels, distinct patterns emerged compared to the initial analysis. For instance, the trend for F2rl1_pS387 became less apparent post normalization, likely attributable to inherent biological variability or the limited sample size. In contrast, the attenuated trends for Hspb1_pS86 and Hspb1_pS15 suggested their initial changes were partly driven by increased total protein abundance. The detailed profiles showing both Δ exp and Δ phosph (normalized) for these phosphoproteins are presented in Supplemental Figure S4.

Lipid metabolism disorders have been demonstrated to play a role in the progression of AKI-to-CKD (61). The phosphoproteins associated with FAO in cluster 5 showed significantly reduced phosphorylation levels at 1 h, 1 day, 3 days, 7 days, and 28 days post-IRI (Fig. 5*B*). These included peroxisomal bifunctional enzyme (Ehhadh, T339 and T341), peroxisomal targeting signal 1 receptor (Pex5, T181), insulin receptor substrate 2 (Irs2, S727), dehydrogenase/reductase SDR family member 6 (Bdh2, S41), and hydroxyacyl-coenzyme A dehydrogenase (Hadh, S13). The phosphorylation levels of these proteins were significantly downregulated after IRI (ANOVA, *p* < 0.05), whereas their total protein expression did not differ significantly among the six groups (ANOVA, *p* > 0.05) (Supplemental Fig. S5). Notably, the phosphorylation sites T339 and T341 in enoyl-CoA hydratase and 3-hydroxyacyl CoA dehydrogenase (Ehhadh) were newly identified, suggesting their potential roles in IRI require further investigation. Ehhadh is one of the enzymes involved in the peroxisomal FAO pathway (62). A previous study has shown that in mice with diabetic kidney disease, loss of Ehhadh exacerbated renal tubular injury and inflammatory infiltration, and led to peroxisome depletion in renal TECs (63). However, the phosphorylation dynamics of Ehhadh at T341 and T339 in response to IRI, as well as their functional implications in AKI-CKD progression, remain unexplored.

### Identification of Dock Family Proteins and Dock2 Knockdown-Mediated Suppression of TEC Proinflammatory Response *In Vitro*

**Fig. 6:**
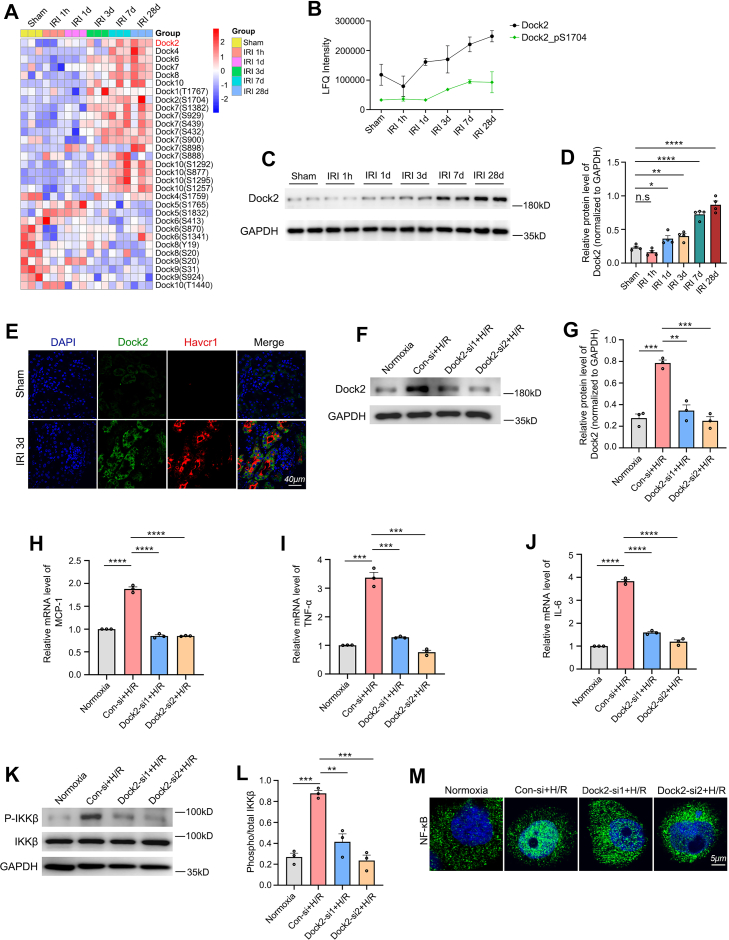
**Dock family proteins are identified, with knockdown of Dock2 demonstrating a significant attenuation of the proinflammatory response of TECs *in vitro*.***A*, heatmap of Dock family proteins and phosphosites (ANOVA, *p* < 0.05). *B*, intensity plots showing Dock2 (*black line*) and its phosphosite S1704 (Dock2_pS1704, *green line*) across the six experimental groups. *C* and *D*, protein expression profiling by western blot (*C*) and subsequent statistical analysis (*D*) of Dock2 in renal cortical samples. *E*, immunofluorescent detection of Dock2 and Havcr1/Kim1 expression and colocalization in renal tissue sections from sham and I/R-treated mice. The scale bar represents 40 μm. *F*, the knockdown efficiency of Dock2 by siRNA in H/R-treated HK-2 cells was analyzed by western blot. *G*, the statistical analysis of (*F*). *H*–*J*, RT-qPCR showing the effects of Dock2 knockdown on mRNA expression levels of MCP-1, TNF-α, and IL-6. *K*, western blot analysis was performed to assess the effects of Dock2 knockdown on IKK-β phosphorylation in H/R-treated HK-2 cells. *L*, densitometric analysis of p-IKKβ from (*K*) was performed with normalization to the respective total protein. *M*, translocation of NF-κB p65 in HK-2 cells was detected by immunofluorescence. NF-κB p65 (*green*), DAPI (*blue*). The scale bar represents 5 μm. Data are presented as mean ± SEM. n.s, not significant; ∗*p* < 0.05; ∗∗*p* < 0.01; ∗∗∗*p* < 0.001; ∗∗∗∗*p* < 0.0001. DAPI, 4′,6-diamidino-2-phenylindole; Dock2, dedicator of cytokinesis 2; Havcr, hepatitis A virus cellular receptor 1; H/R, hypoxia/reoxygenation; IKKβ, IκB kinase beta; Kim1, kidney injury molecule-1; IL-6, interleukin-6; MCP-1, monocyte chemoattractant protein-1; RT-qPCR, reverse transcription quantitative PCR; TEC, tubular epithelial cell; TNF-α, tumor necrosis factor-α.

Of interest, through our proteomic and phosphoproteomic analyses, we identified Dock protein family, a family of Rho guanine nucleotide exchange factors, with 11 members (64). They regulate migration, proliferation, and adhesion and have been involved in various pathologies, including cancer and defects in the central nervous and immune system (64, 65). However, the functional roles of the Dock family in renal pathology remain poorly characterized. Ten Dock proteins and a total of 57 phosphosites in this family were identified in our study, but not all of them showed significant differences after IRI. A heatmap was plotted to obtain an overview of the significantly changed Dock proteins and/or their phosphorylated forms (ANOVA, *p* < 0.05) (Fig. 6*A*).

Inflammation is a well-established driver of AKI-to-CKD progression (56). Among Dock family proteins, Dock2 is of particular interest due to its demonstrated proinflammatory roles in atherosclerosis, spinal cord injury, and endotoxemia-induced acute lung injury (66, 67, 68). However, its specific function in IRI-induced AKI and subsequent CKD transition remains unexplored. Our proteomic and phosphoproteomic analyses revealed that the expression level of Dock2 increased gradually from 1 day to 28 days post-IRI, while its phosphorylated form (pS1704) showed only a fractional increase (Fig. 6*B*). Statistical analysis for the Δ exp and Δ phosph (normalized) indicated that the increase in Dock2 protein was significant at 7 days and 28 days post-IRI, whereas its normalized phosphorylation at S1704 did not reach statistical significance (ANOVA, *p* > 0.05), as detailed in Supplemental Figure S6. To biochemically validate this observed upregulation in Dock2 protein expression, we performed western blot analysis, which confirmed a time-dependent, significant upregulation of Dock2 protein expression at 1 day, 3 days, 7 days, and 28 days post-IRI compared to sham controls (Fig. 6, *C* and *D*). Moreover, immunofluorescent staining of Dock2 and Havcr1/Kim1 (a tubular injury biomarker) in kidney tissues post-IRI revealed significantly elevated Dock2 expression in the IRI group compared to sham controls, with predominant localization to injured TECs (Fig. 6*E*). To investigate the role of Dock2 in TECs during IRI, we designed two Dock2-specific siRNAs. First, we confirmed that both siRNAs effectively knocked down Dock2 under standard normoxic conditions (Supplemental Fig. S7). We then established an *in vitro* H/R model to mimic IRI. As shown in Figure 6, *F* and *G*, H/R treatment significantly upregulated Dock2 protein expression in HK-2 cells, while transfection with Dock2-specific siRNAs effectively knocked down Dock2 protein levels under H/R conditions. Next, we examined the role of Dock2 in the proinflammatory response of HK-2 cells. RT-qPCR showed that Dock2 knockdown significantly suppressed H/R-induced expression of MCP-1, TNF-α, and IL-6 mRNA (Fig. 6, *H*–*J*). NF-κB activation critically regulates the expression of the above proinflammatory mediators (68, 69), primarily through the canonical pathway mediated by the IκB kinase (IKK) complex, the most common and well-characterized mechanism of NF-κB regulation (69). Specifically, the IKK complex (IKKα/IKKβ/IKKγ) activates canonical NF-κB signaling, through IKKβ-mediated phosphorylation of IκBα. This triggers IκBα ubiquitination and degradation, releasing NF-κB (typically p50/P65 dimers) for nuclear translocation and transcriptional induction of proinflammatory cytokines (69). In HK-2 cells subjected to H/R, we observed significant phosphorylation of IKKβ. However, Dock2 knockdown markedly attenuated this H/R-induced IKKβ phosphorylation (Fig. 6, *K* and *L*). Furthermore, we assessed NF-κB nuclear translocation by immunofluorescence staining of HK-2 cells using DAPI and an anti-NF-κB p65 antibody. H/R treatment significantly enhanced NF-κB nuclear translocation compared to normoxic conditions. However, this H/R-induced nuclear translocation was substantially suppressed in Dock2-knockdown cells (Fig. 6*M*). Collectively, these results demonstrate that Dock2 knockdown significantly suppresses the proinflammatory response in HK-2 cells induced by H/R, at least in part through inhibition of IKKβ phosphorylation and subsequent blockade of NF-κB nuclear translocation.

### Pharmacological Inhibition of Dock2 by CPYPP Attenuates Tubular Injury, Inflammation, and Fibrosis After Renal IRI

**Fig. 7:**
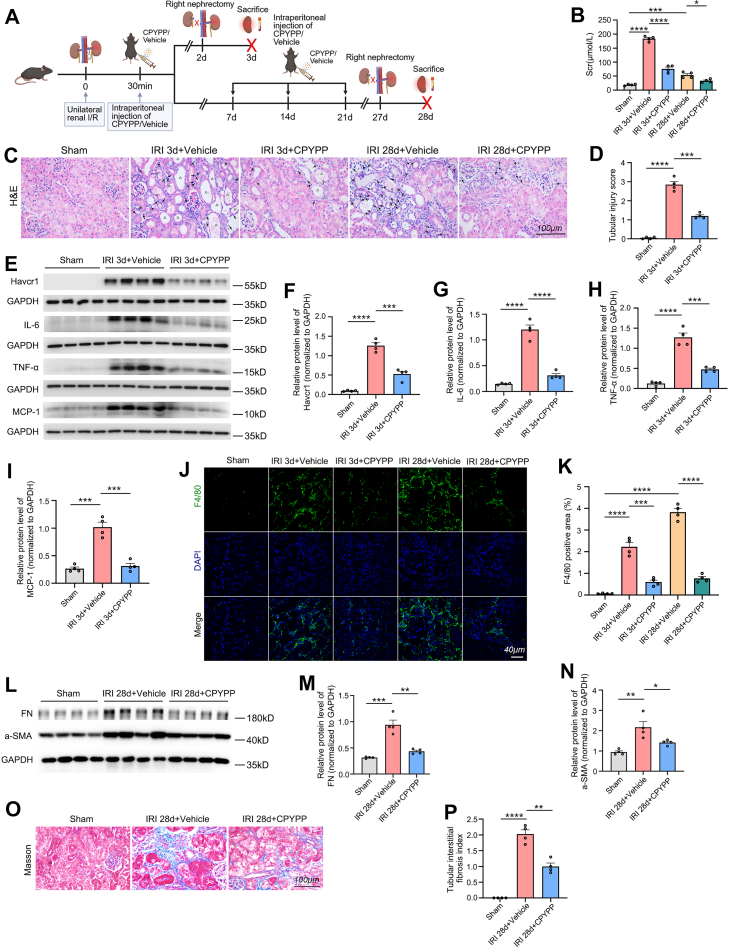
**Inhibition of Dock2 by CPYPP attenuates tubular injury, inflammatory infiltration and interstitial fibrosis after renal IRI in mice.***A*, schematic of the experimental design for unilateral I/R in mice treated with CPYPP or vehicle via i.p. injection following I/R induction. *B*, measurement of Scr levels. *C*, H&E staining was performed to assess the effect of pharmacological inhibition of Dock2 by CPYPP on renal tissue architecture in mice at 3 days and 28 days post-I/R. Inflammatory cells, tubular casts, and dilated tubules are indicated by *solid arrows*, *hollow arrows*, and *triangles*, respectively. The scale bar represents 100 μm. *D*, tubular injury score at 3 days post-I/R (quantified from *C*). *E*–*I*, western blot (*E*) and quantitative analysis (*F*–*I*) of renal cortical Havcr1/Kim1, IL-6, TNF-α, and MCP-1 expression in sham-operated and 3 days post-I/R mice, with or without CPYPP treatment. *J*, immunofluorescent staining for the macrophage marker F4/80 in renal cortical sections to assess inflammatory infiltration. The scale bar represents 40 μm. *K*, the quantitative analysis of the F4/80 immunofluorescence staining from (*J*). *L*–*N*, western blot (*L*) and quantitative analysis (*M* and *N*) of renal cortical FN and α-SMA expression in sham-operated and 28 days post-I/R mice, with or without CPYPP treatment. *O*, Masson's trichrome staining was used to evaluate the effect of CPYPP on renal collagen deposition at 28 days post-I/R, with quantification data shown in (*P*). The scale bar represents 100 μm. Data are presented as mean ± SEM. n.s, not significant; ∗*p* < 0.05; ∗∗*p* < 0.01; ∗∗∗*p* < 0.001; ∗∗∗∗*p* < 0.0001. CPYPP, 4-[3′-(2″-chlorophenyl)-2′-propen-1′-ylidene]-1-phenyl-3,5-pyrazolidinedione; Dock2, dedicator of cytokinesis 2; FN, fibronectin; Havcr, hepatitis A virus cellular receptor 1; IL-6, interleukin-6; IRI, ischemia-reperfusion injury; Kim1, kidney injury molecule-1; MCP-1, monocyte chemoattractant protein-1; α-SMA, alpha-smooth muscle actin; Scr, serum creatinine; TNF-α, tumor necrosis factor-α.

We next assessed the effect of CPYPP, a DOCK2 small-molecule inhibitor, on renal IRI in mice, using the experimental design shown in Figure 7*A*. Compared with vehicle-treated IRI controls, CPYPP treatment significantly reduced Scr levels at both 3 days and 28 days post-IRI (Fig. 7*B*). H&E staining was performed to evaluate renal tissue injury. At 3 days post-IRI, CPYPP treatment attenuated acute damage including brush border loss, tubular dilation, cast formation, and inflammatory cell infiltration (Fig. 7, *C* and *D*). By 28 days post-IRI, CPYPP reduced chronic injury features such as tubular atrophy, interstitial expansion and persistent inflammatory cell infiltration (Fig. 7*C*). Western blot further demonstrated that CPYPP administration significantly downregulated the protein expression of tubular injury marker Havcr1/Kim1 and proinflammatory mediators (IL-6, TNF-α, and MCP-1) at 3 days post-IRI (acute phase) (Fig. 7, *E*–*I*). Consistent with the changes of these proinflammatory mediators, immunofluorescence staining revealed that CPYPP markedly reduced F4/80^+^ macrophage density in the renal cortex at 3 days post-IRI (Fig. 7, *J* and *K*), demonstrating its anti-inflammatory effect in I/R-AKI. At 28 days post-IRI (chronic phase), CPYPP further suppressed macrophage accumulation (Fig. 7, *J* and *K*), indicating a persistent anti-inflammatory effect in I/R-CKD. Renal fibrosis represents a key pathological feature in the progression from AKI to CKD (70). To investigate whether CPYPP ameliorates renal fibrosis after IRI, we analyzed fibrotic markers by western blot. The results showed that fibronectin (FN) and alpha-smooth muscle actin (α-SMA) protein levels were significantly elevated at 28 days post-IRI, but CPYPP treatment markedly reduced their expression (Fig. 7, *L*–*N*). To further assess renal collagen deposition, we performed Masson's trichrome staining. Histological analysis revealed a remarkable increase in tubulointerstitial fibrosis at 28 days post-IRI. Notably, CPYPP treatment effectively attenuated this fibrotic response (Fig. 7, *O* and *P*).

Taken together, these findings demonstrate that Dock2 inhibition by CPYPP significantly attenuates tubular injury, inflammation, and fibrosis in mice following renal IRI, suggesting that targeting Dock2 may represent a therapeutic strategy to mitigate AKI-to-CKD progression.

## Discussion

Given that IRI is a well-established trigger for AKI-to-CKD progression, a comprehensive elucidation of the global molecular mechanisms driving this pathological transition is critically important for the development of novel therapeutic strategies. Using global proteomic and phosphoproteomic profiling in a murine IRI model, we systematically mapped dynamic changes in protein expression, phosphorylation events, and key signaling pathways implicated in the AKI-to-CKD transition.

Our study demonstrated that Havcr1/Kim1 expression was significantly upregulated at 1 day and 3 days post-IRI (peaking at 3 days), followed by a progressive decline at 7 days and 28 days. This tissue-level expression pattern parallels the kinetic profile observed in serum and urine Havcr1/Kim1 levels reported in a previous study, in which peak concentrations occurred during the acute phase followed by gradual decline post-IRI (71). These collective findings from both tissue and biofluid analyses strongly support Havcr1/Kim1 as a reliable biomarker for AKI detection and monitoring (72). However, both IL-6 and F4/80^+^ macrophage infiltration exhibited sustained elevation throughout all examined time points (1 day to 28 days), indicating persistent inflammatory activation spanning both acute and chronic phases of IRI. This finding is consistent with the previous study's report that severe AKI can lead to maladaptive repair and sustained tubulointerstitial inflammation (71). Sustained immune activation mediated by injured tubules is a key mechanism underlying chronic inflammation in maladaptive renal repair. Specifically, after injury, TECs acquire an activated secretory phenotype, releasing proinflammatory chemokines that actively recruit immune cells to sites of renal tissue damage (21). For instance, injured proximal tubule cells significantly upregulate MCP-1 production, which plays an important role in recruiting monocytes and their derived macrophages during interstitial inflammation in CKD progression (73). Furthermore, activated TECs secrete a broad spectrum of inflammatory cytokines, including interleukins (e.g. IL-6, IL-1β) and TNF-α, which amplify local inflammatory responses and perpetuate tubular injury (74, 75). In addition, tubular cells can also communicate with immune cells through exosome-mediated mechanisms. For example, exosomes derived from albumin-exposed TECs have been shown to deliver MCP-1 mRNA to macrophages, triggering both inflammatory activation and chemotactic migration of these immune cells (76). Chronic inflammation is now widely recognized as a critical determinant of AKI-to-CKD progression (56). A more precise elucidation of its underlying mechanisms will not only resolve key pathophysiological uncertainties in this field but also accelerate the development of targeted therapeutic strategies to interrupt this maladaptive transition.

Our proteomic and phosphoproteomic data consistently revealed the enrichment and sustained activation of NF-κB signaling across the AKI-to-CKD transition, as evidenced by the persistent upregulation of NF-κB-associated proteins and increased phosphorylation signals within this pathway. The NF-κB pathway represents one of the key intracellular signaling cascades activated after inflammation (77). When TECs are injured, they secrete damage-associated molecular patterns (DAMPs) such as heat-shock proteins and high mobility group box 1 (HMGB1), a nonhistone chromatin-binding protein, which subsequently engage toll-like receptors (TLRs), particularly TLR4, TLR2, and TLR7 (78). Ligand binding to these TLRs initiates downstream signaling events that ultimately converge on NF-κB activation (3). Upon activation, the NF-κB pathway induces the expression of proinflammatory mediators, including cytokines like TNF-α, IL-6, and IL-1, as well as chemokines such as MCP-1 and CXCL2 (69, 79). This pathway is essential to orchestrating the immune response, underscoring its crucial role in inflammation (77). Beyond its proinflammatory effects, the NF-κB pathway contributes to renal fibrosis through multiple mechanisms. In diabetic nephropathy, NF-κB promotes tubular epithelial to mesenchymal transition (EMT) via the NLRP3 inflammasome/caspase-1/IL-1β axis (80), while in aristolochic acid-induced kidney injury, it facilitates fibrosis through the miR-382/PTEN/AKT pathway (81). Experimental evidence further indicates that sustained NF-κB activation participates in fibrosis development following IRI (82), highlighting its involvement in CKD progression after AKI. Given the key role of sustained NF-κB activation in driving renal inflammation and fibrosis across AKI-CKD progression, targeted inhibition of this pathway emerges as a compelling therapeutic imperative.

To experimentally validate the functional contribution of NF-κB to the sustained inflammatory phenotype, we inhibited p65 nuclear translocation, effectively reducing it to levels comparable to the sham group. This intervention markedly suppressed, but did not abolish, the upregulation of IL-6 post-IRI. These findings indicate that the sustained upregulation of IL-6 is predominantly, but not exclusively, mediated by the NF-κB pathway. The residual IL-6 expression implicates the involvement of alternative signaling mechanisms, with the MAPK pathway serving as a potential contributor, as reported to mediate IL-6 production independently of NF-κB in another stress model (83).

Lipid metabolism dysregulation has been implicated in the pathogenesis of AKI-to-CKD transition (61). Our findings identified impaired FAO during AKI-to-CKD progression, as indicated by persistent downregulation of FAO-related proteins and decreased phosphorylation levels in this pathway. This observation aligns with a prior proteomic study showing FAO deficiency in post ischemic kidneys at 3 days, 7 days, and 21 days following IRI (84). Given that FAO serves as the primary energy source for most renal tubule segments (61), its suppression during AKI (85) may contribute to sustained metabolic dysfunction. Notably, when exposed to pathological stressors such as IRI, TECs develop prolonged FAO suppression that persists well beyond the acute injury phase (86). Failure to restore FAO post injury may lead to lipid accumulation and metabolic reprogramming in TECs, promoting EMT and elevating CKD risk even after transient AKI (87). Moreover, intracellular lipid overload exacerbates tubular injury and interstitial fibrosis by triggering proinflammatory responses (e.g. TNF-α, MCP-1, and IL-6) and excessive reactive oxygen species production (86). Collectively, these findings highlight the therapeutic potential of early FAO restoration in TECs to mitigate AKI severity and prevent CKD progression.

It is worth noting that our interpretation of phosphoproteomic dynamics was further complemented by an analysis of Δ exp and Δ phosph (normalized), which distinguishes phosphorylation-specific regulation from changes in total protein abundance. For instance, the sustained downregulation of Ctnnd1_pS288 after normalization confirms a true posttranslational event. In contrast, for phosphosites such as F2rl1_pS387, the observed trends were attenuated post normalization, likely constrained by biological variability and limited sample size. For others, including Hspb1_pS86 and Hspb1_pS15, the attenuated trends indicate that their initial phosphorylation changes were partly driven by increased total protein abundance. Importantly, the unnormalized phospho-signal for these proteins retains biological relevance because it reflects the absolute abundance of the activated, phosphorylated proteins, which is a key factor influencing downstream signaling. Therefore, concurrent increases in both total protein expression and phosphorylation may synergistically drive the functional output of signaling pathways.

In this context, we focused on Dock2, a member of the Dock family, for several compelling reasons. Although recent work implicated Dock2 in activating EMT during renal fibrosis via the Rac1/PI3K/AKT pathway in a murine unilateral ureteral obstruction (UUO) model (88), its role in IRI-induced AKI and the subsequent transition to CKD remains unexplored. Notably, Dock2 exhibited validated and persistent upregulation from 1 day through 28 days after IRI, was specifically localized to injured TECs, and had reported linkages to NF-κB signaling in a model of endotoxemia-induced acute lung injury (68). Its reported proinflammatory roles in other pathologies further supported its potential relevance. Moreover, the availability of a selective pharmacological inhibitor (CPYPP) enabled direct functional validation. These features collectively nominated Dock2 as a compelling candidate for mechanistic investigation in the AKI-to-CKD continuum.

Dock2 expression was significantly upregulated post-IRI and was predominantly localized to injured TECs. However, the precise molecular mechanisms governing Dock2 upregulation in this context remain to be fully elucidated. Given the proinflammatory roles of Dock2 in some pathologies including spinal cord injury, atherosclerosis, and endotoxemia-induced acute lung injury (66, 67, 68), we investigated its potential involvement in regulating inflammation during I/R-AKI and CKD progression. Our findings demonstrated that genetic knockdown of Dock2 attenuated proinflammatory responses in TECs subjected to H/R *in vitro*. Consistently, pharmacological inhibition of Dock2 using CPYPP mitigated inflammation in both AKI and CKD phases following IRI *in vivo*. NF-κB signaling is a well-established pathway driving inflammatory responses (77). Mechanism exploration revealed that Dock2 knockdown suppressed IKKβ phosphorylation and NF-κB nuclear translocation. As a guanine exchange factor for Rac family small GTPase 1 (Rac1), Dock2 has been documented to regulate Rac1 activity (89). Therefore, further studies are needed to determine whether Rac1 participates in Dock2-regulated inflammation in I/R-induced renal injury. Furthermore, Dock2 has been shown to promote adipose tissue expansion by suppressing FAO, lipolysis, and thermogenesis (90). Intracellular lipid accumulation in renal TECs is a known trigger for proinflammatory responses (86). Therefore, determining whether Dock2 drives lipid metabolic dysregulation and subsequent inflammation in renal IRI represents an important avenue for future research. To our knowledge, this is the first study to reveal the functional role and molecular mechanism of Dock2 in driving AKI-to-CKD progression post-IRI.

Although our study provides novel insights, it is not without limitations. One limitation is that our proteomic and phosphoproteomic analyses were performed in a murine renal IRI model, not human samples. Although interspecies differences exist, rodent models of renal IRI induced by blockage of blood flow to kidneys have been well-established as valuable tools for simulating human renal IRI and are widely adopted as experimental systems for investigating AKI-to-CKD transition (91). Another limitation concerns the inherent technical constraints of untargeted proteomics. Specifically, the absence of certain low-abundance cytokines (e.g. IL-6, TNF-α, and MCP-1) and specific phosphorylation events (e.g. on IKKβ) in the MS data reflects these limitations: the signals of low-abundance proteins can be masked by high-abundance proteins in complex tissue lysates, while phosphorylation sites with low stoichiometry or those on peptides with properties challenging for MS detection (e.g. poor ionization or fragmentation) often evade identification. Nevertheless, these key targets were robustly validated using targeted methods (western blot and/or RT-qPCR). An additional limitation is the lack of experimental validation for the phosphoproteomics findings, attributable to the unavailability of phospho-specific antibodies targeting the identified sites.

In summary, our proteomic and phosphoproteomic profiling deciphered molecular dynamics of AKI-to-CKD progression post-IRI. Sustained activation of NF-κB signaling and impaired FAO was revealed. Moreover, Dock2 was demonstrated to be a key mediator of proinflammatory responses in TECs via the IKKβ/NF-κB pathway. These findings nominate Dock2 as a therapeutic target to mitigate AKI-to-CKD progression.

## Data Availability

The mass spectrometry proteomics and phosphoproteomics data have been deposited to the ProteomeXchange Consortium via the iProX partner repository (92) (accession: PXD065974, https://proteomecentral.proteomexchange.org).

## Supplementary Data

This article contains supplemental data.

## Conflict of Interest

The authors declare no competing interests.
