# Supplementary material for: Temporal Proteomic and Phosphoproteomic Profiling Deciphers Molecular Dynamics of Acute-to-Chronic Kidney Disease After Ischemia-Reperfusion Injury, With Dock2 Emerging as a Key Regulator
Source: Mol Cell Proteomics. 2026 Jan 12;25(2):101509. doi: 10.1016/j.mcpro.2026.101509 (PMC12915234; doi:10.1016/j.mcpro.2026.101509)
Supplement: Supplemental Data [file mmc13.docx]

**Supplemental Information for**

**Temporal Proteomic and Phosphoproteomic Profiling Deciphers Molecular Dynamics of Acute-to-Chronic Kidney Disease After Ischemia-Reperfusion Injury, With Dock2 Emerging as a Key Regulator**

Shaowu Zhang^1,2^, Huasheng Luo^1^, Miaotao Wei^1^, Yanmei Yu^1^, Hongluan Wu^1^, Tongtong Ma^3^, Minjie Zhang^1^, Huafeng Liu^1,2,^*, Peng Wang^1,^*

^1^Guangdong Provincial Key Laboratory of Autophagy and Major Chronic Non-Communicable Diseases, Key Laboratory of Prevention and Management of Chronic Kidney Diseases of Zhanjiang City, Institute of Nephrology, Affiliated Hospital of Guangdong Medical University, No. 57, South Renmin Avenue, Xiashan District, Zhanjiang, Guangdong 524023, China

^2^Dr. Neher's Biophysics Laboratory for Innovative Drug Discovery, State Key Laboratory of Quality Research in Chinese Medicine, Faculty of Chinese Medicine, Macau University of Science and Technology, Avenida Wai Long, Taipa, Macau 999078, China

^3^Department of Anesthesiology, Affiliated Hospital of Guangdong Medical University, Zhanjiang, Guangdong, China

*Corresponding authors

Huafeng Liu, Email: [liuhf@gdmu.edu.cn](mailto:liuhf@gdmu.edu.cn); Peng Wang, Email: wangpeng@gdmu.edu.cn

**Running title:** Multi-proteomic Profiling of AKI-to-CKD Post-IRI

**1.** **Supplemental Figures**

**Figure S1.** Inhibition of NF-κB p65 nuclear translocation markedly suppresses, but does not abolish, IL-6 upregulation post-IRI.

**Figure S2.** Venn diagram illustrating the overlap between proteins identified in the global proteome and phosphoproteome.

**Figure S3.** Temporal profiles of changes in normalized phosphorylation and changes in total protein expression for the overlapping phosphosites.

**Figure S4.** Temporal profiles of changes in normalized phosphorylation and changes in total protein expression for the phosphoproteins enriched in the I-kappaB kinase/NF-kappaB signaling pathway in cluster 3 of Fig. 5*B*.

**Figure S5.** Intensity plots of the phosphoproteins enriched in the fatty acid beta-oxidation in cluster 5 of Fig. 5B and their corresponding total proteins.

**Figure S6.** Temporal profiles of changes in normalized phosphorylation and changes in total protein expression for Dock2_pS1704.

**Figure S7.** Validation of the knockdown efficiency of Dock2 siRNAs in HK-2 cells under normoxic conditions.

1. **Supplemental Tables (separate files)**

**Supplemental Table S1.** List of all identified proteins and their quantification data.

**Supplemental Table S2.** List of identified proteins qualified for differential expression analysis in proteomics.

**Supplemental Table S3.** List of differentially expressed proteins (DEPs) in renal IRI at 1d, 3d, 7d, and 28d compared to sham.

**Supplemental Table S4.** List of overlapping DEPs across IRI 1d, 3d, 7d, and 28d vs. sham.

**Supplemental Table S5.** GO biological process enrichment of overlapping upregulated DEPs in renal IRI.

**Supplemental Table S6.** GO biological process enrichment of overlapping downregulated DEPs in renal IRI.

**Supplemental Table S7.** GO biological process enrichment results for the six distinct time-dependent protein expression clusters.

**Supplemental Table S8.** Table of all high-confidence phosphorylation sites and their quantification data.

**Supplemental Table S9.** Peptide identification table–phosphoproteome.

**Supplemental Table S10.** List of differentially detected phosphosites in renal IRI at 1h, 1d, 3d, 7d, and 28d vs. sham.

**Supplemental Table S11.** List of overlapping differentially expressed phosphosites among comparisons: IRI 1 h vs. sham, IRI 1 d vs. sham, IRI 3 d vs. sham, IRI 7 d vs. sham, and IRI 28 d vs. sham.

**Supplemental Table S12.** GO biological process enrichment of the seven time-dependent phosphoprotein clusters post-IRI.

**Supplemental Figure 1**


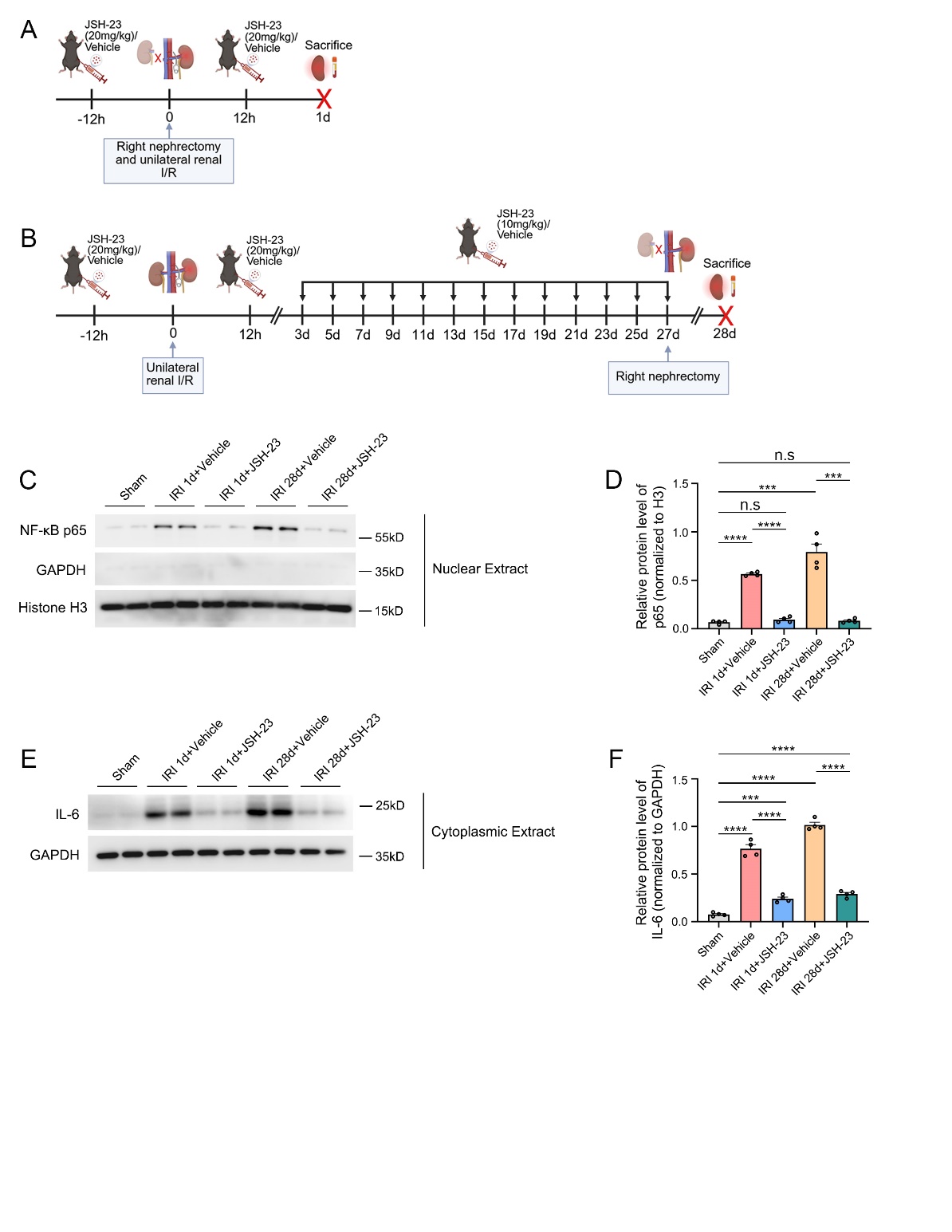


**Figure S1. Inhibition of NF-κB p65 nuclear translocation markedly suppresses, but does not abolish, IL-6 upregulation post-IRI.** *A*–*B*, Schematics of JSH-23 or vehicle administration via i.p. injection for experiments terminated at 1 d (*A*) or 28 d (*B*) post-IRI. *C*–*D***,** Western blot (*C*) and quantification (*D*) of NF-κB p65 levels in renal cortical nuclear extracts from the indicated experimental groups. Histone H3 was used as the nuclear loading control. The negligible GAPDH signal confirmed the high purity of the nuclear fraction. *E*–*F*, Western blot (*E*) and quantification (*F*) of IL-6 protein levels in renal cortical cytoplasmic extracts from the indicated experimental groups. GAPDH was used as the cytoplasmic loading control. Data are presented as mean ± SEM. n.s, not significant; ***p < 0.001; ****p < 0.0001.

**Supplemental Figure 2**


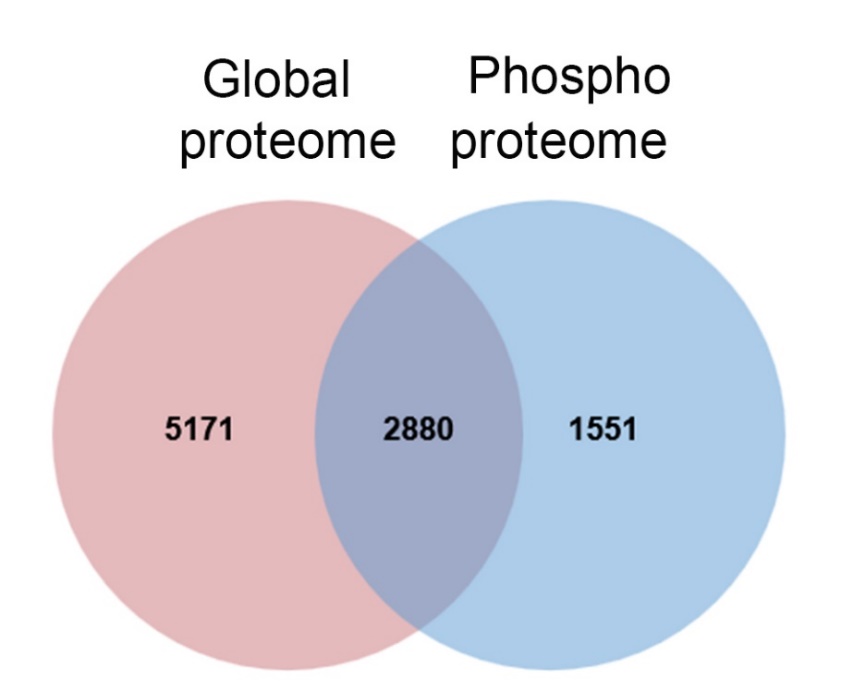


**Figure S2. Venn diagram illustrating the overlap between proteins identified in the global proteome and phosphoproteome.**

**Supplemental Figure 3**


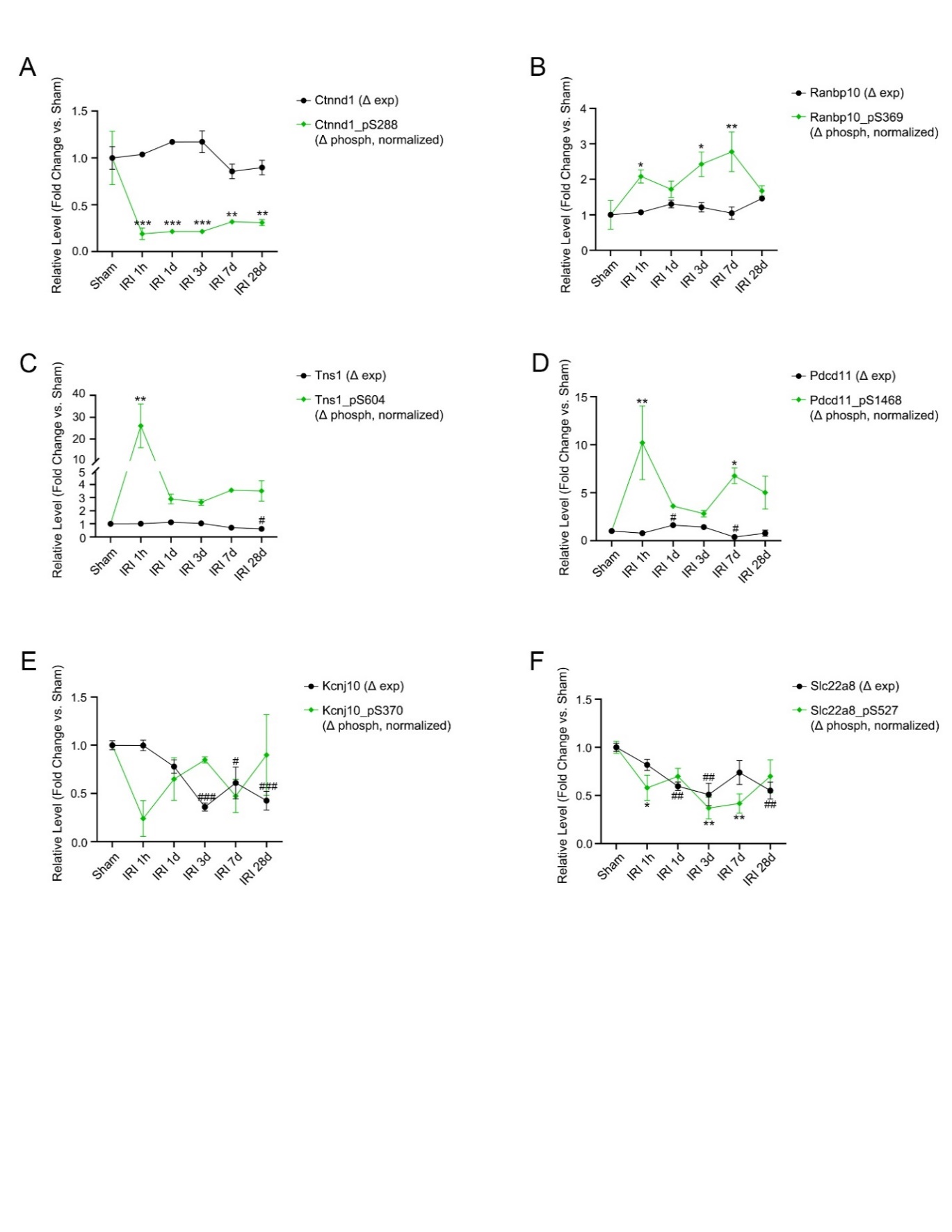


**Figure S3.** **Temporal profiles of changes in normalized phosphorylation and changes in total protein expression for the overlapping phosphosites.** *A*–*F*, Temporal dynamics of the overlapping phosphosites (see Fig. 4*A*): Ctnnd1_pS288 (*A*); Ranbp10_pS369 (*B*); Tns1_pS604 (*C*); Pdcd11_pS1468 (*D*); Kcnj10_pS370 (*E*); Slc22a8_pS527 (*F*). In each panel, the green line and black line represent the change in normalized phosphorylation (Δ phosph, normalized) and the corresponding change in total protein expression (Δ exp), respectively. Normalization was performed by dividing the phosphorylation intensity by its matched total protein intensity within each biological replicate. All plotted values are expressed as fold change relative to the mean of the sham group. Data are presented as mean ± SEM. Statistical significance versus sham is indicated as: Δ phosph (normalized): *p < 0.05, **p < 0.01, ***p < 0.001; Δ exp: #p < 0.05, ##p < 0.01, ###p < 0.001.

**Supplemental Figure 4**


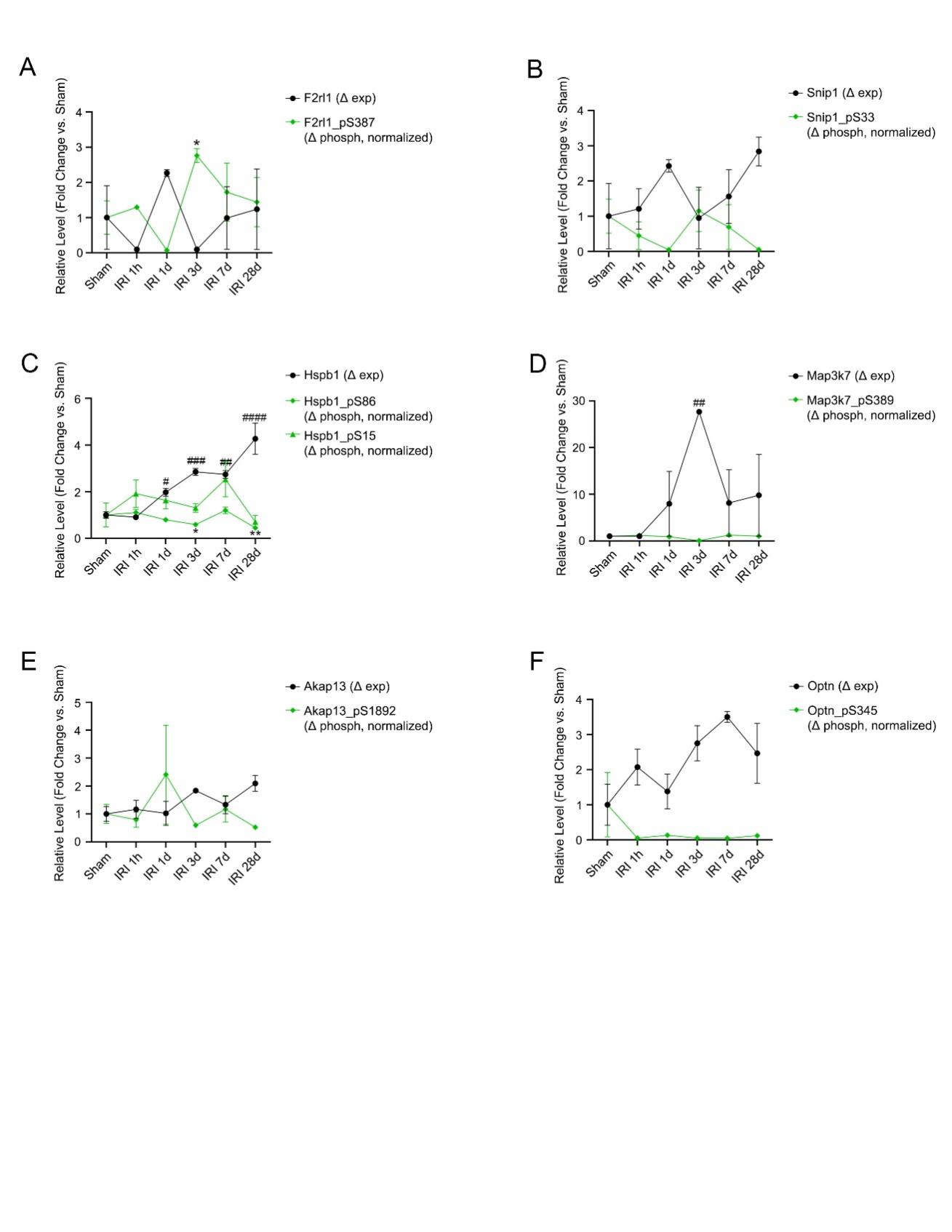


**Figure S4. Temporal profiles of changes in normalized phosphorylation and changes in total protein expression for the phosphoproteins enriched in the I-kappaB kinase/NF-kappaB signaling pathway in cluster 3 of Fig. 5*B*.** *A*–*F*, Temporal dynamics of the phosphosites: F2rl1_pS387 (*A*); Snip1_pS33 (*B*); Hspb1_pS15 and Hspb1_pS86 (*C*); Map3k7_pS389 (*D*); Akap13_pS1892 (*E*); Optn_pS345 (*F*). In each panel, the green line and black line represent the change in normalized phosphorylation (Δ phosph, normalized) and the corresponding change in total protein expression (Δ exp), respectively. Normalization was performed by dividing the phosphorylation intensity by its matched total protein intensity within each biological replicate. All plotted values are expressed as fold change relative to the mean of the sham group. Data are presented as mean ± SEM. Statistical significance versus sham is indicated as: Δ phosph (normalized): *p < 0.05, **p < 0.01; Δ exp: #p < 0.05, ##p < 0.01, ###p < 0.001, ####p < 0.0001.

**Supplemental Figure 5**


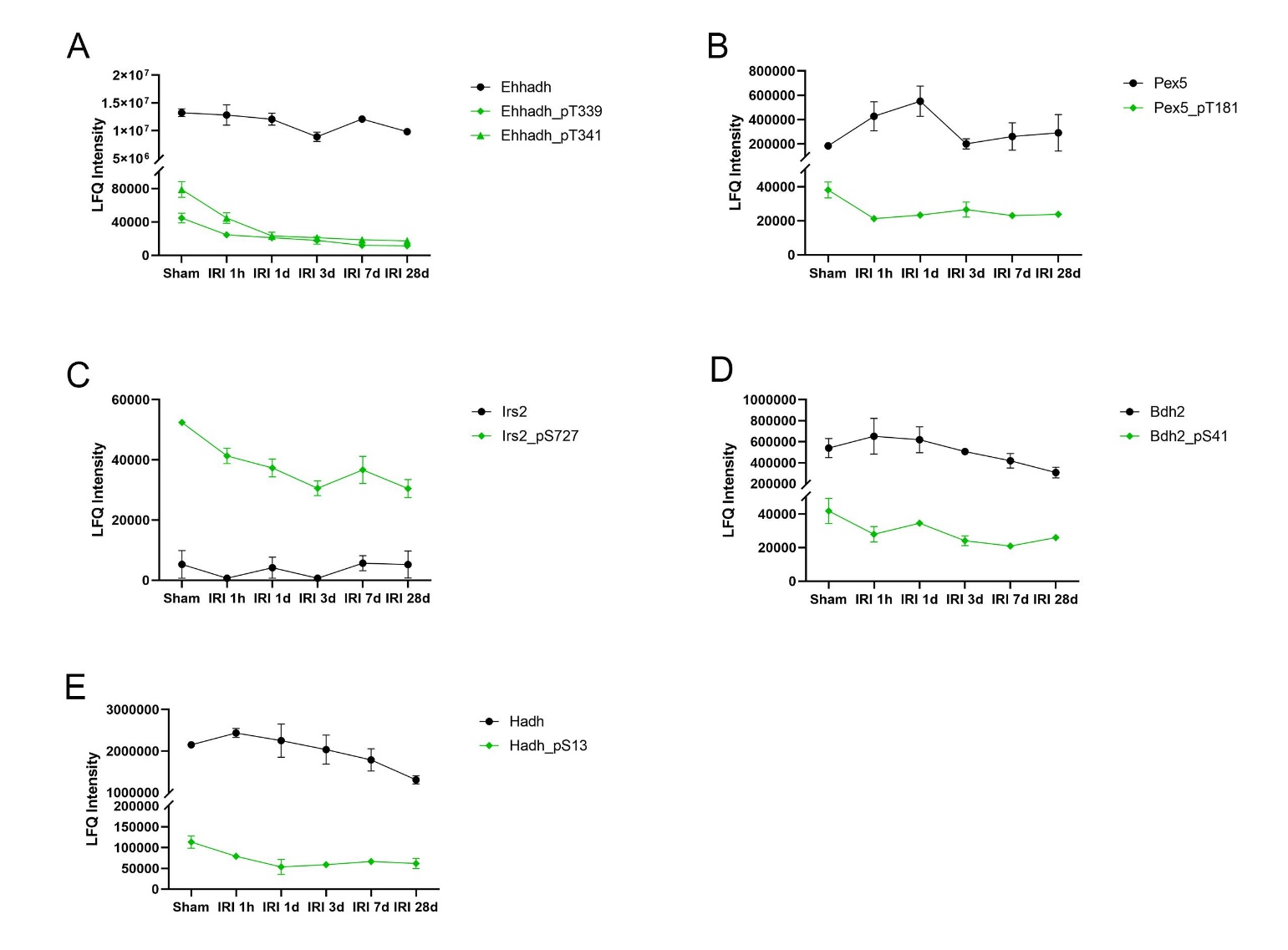


**Figure S5. (*A*****–*E*) Intensity plots of the phosphoproteins (green line) enriched in the fatty acid beta-oxidation in cluster 5 of Fig. 5*B* and their corresponding total proteins (black line).**

**Supplemental Figure 6**


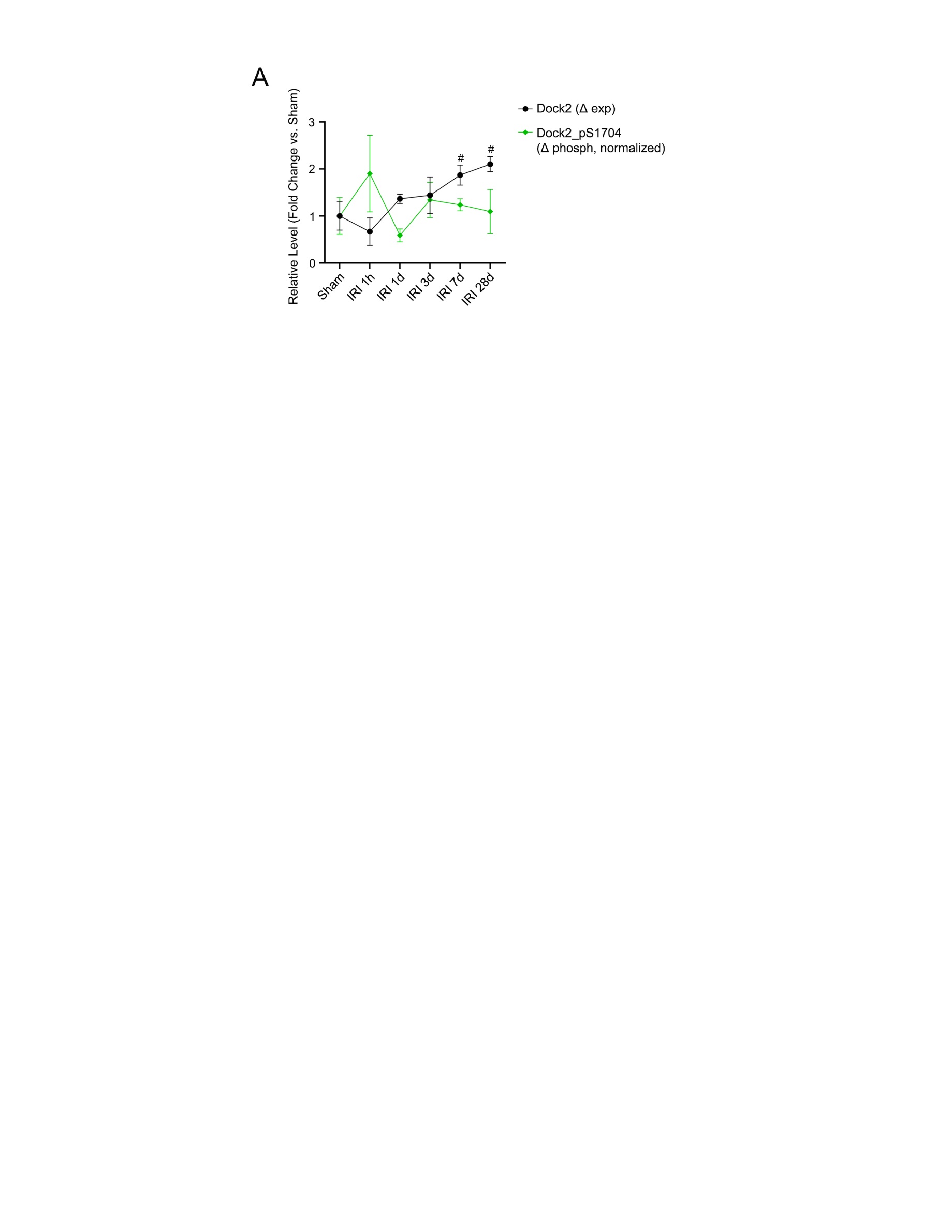


**Figure S6. Temporal profiles of changes in normalized phosphorylation and changes in total protein expression for Dock2_pS1704.** The green line and black line represent the change in normalized phosphorylation (Δ phosph, normalized) and the corresponding change in total protein expression (Δ exp), respectively. Normalization was performed by dividing the phosphorylation intensity by its matched total protein intensity within each biological replicate. All plotted values are expressed as fold change relative to the mean of the sham group. Data are presented as mean ± SEM. Statistical significance versus sham is indicated as: Δ exp: #p < 0.05.

**Supplemental Figure 7**


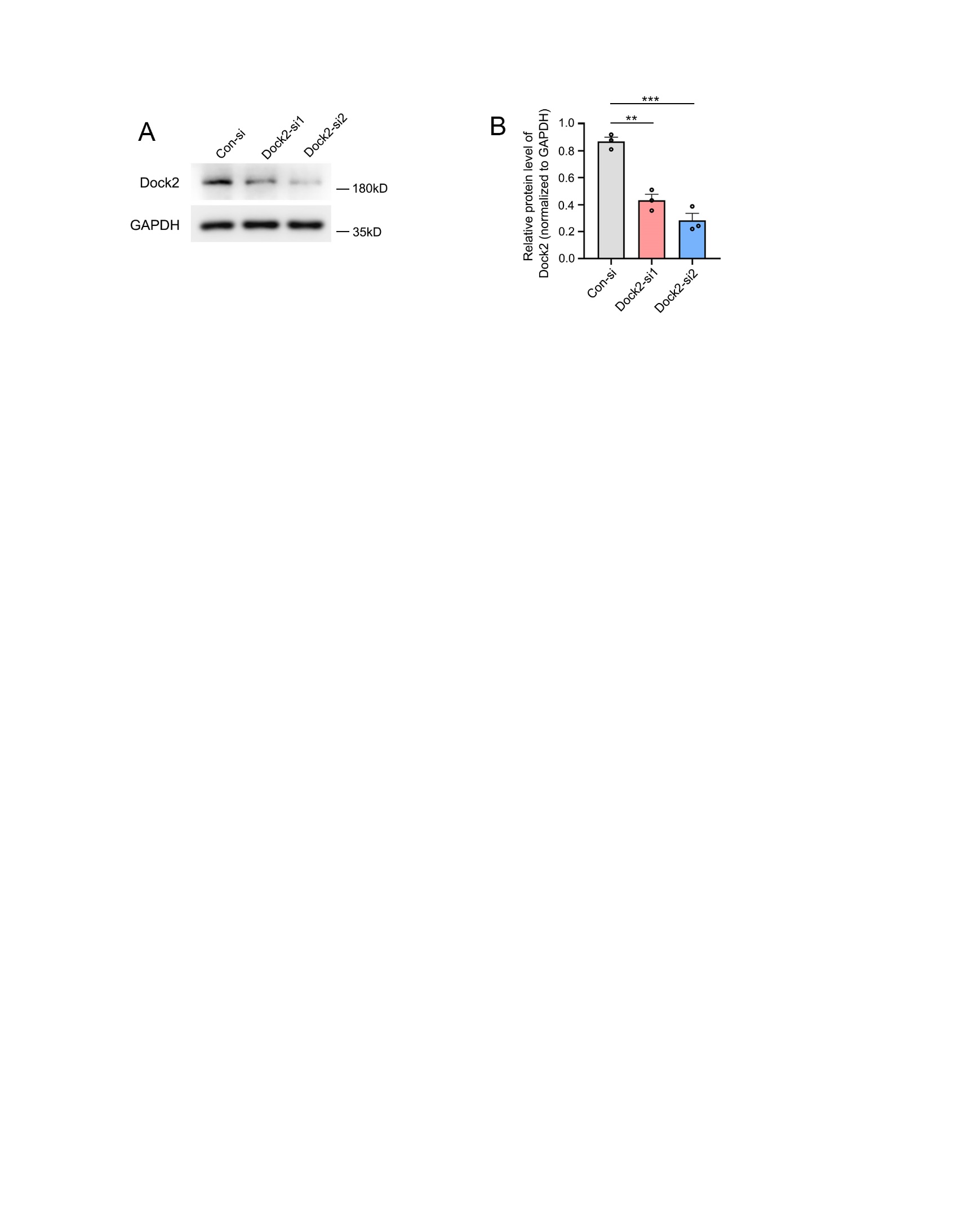


**Figure S7. Validation of the knockdown efficiency of Dock2 siRNAs in HK-2 cells under normoxic conditions.** *A*, Representative western blot showing Dock2 protein levels in HK-2 cells transfected with control siRNA (Con-si), Dock2-si1, or Dock2-si2 under normoxia. *B*, Quantification of Dock2 protein levels normalized to GAPDH. Data are presented as mean ± SEM. **p < 0.01; ***p < 0.001.
